# Transcriptome analysis of oil palm inflorescences revealed candidate genes for an auxin signaling pathway involved in parthenocarpy

**DOI:** 10.7717/peerj.5975

**Published:** 2018-12-17

**Authors:** Suthasinee Somyong, Kitti Walayaporn, Nukoon Jomchai, Chaiwat Naktang, Tanapong Yodyingyong, Chalermpol Phumichai, Wirulda Pootakham, Sithichoke Tangphatsornruang

**Affiliations:** 1 National Center for Genetic Engineering and Biotechnology (BIOTEC), National Science and Technology Development Agency, Pathum Thani, Thailand; 2 Interdisciplinary Graduate Program in Genetic Engineering and Bioinformatics, Kasetsart University, Bangkok, Thailand; 3 Department of Agronomy, Faculty of Agriculture, Kasetsart University, Bangkok, Thailand

**Keywords:** RNA seq, Parthenocarpy, Oil palm, Auxin

## Abstract

Oil palm parthenocarpic fruits, which are produced without fertilization, can be targeted to increase oil content because the majority of the fruit is occupied by mesocarp, the part in which palm oil is stored. Consequently, gaining an understanding of the parthenocarpic mechanism would be instrumental for producing parthenocarpic oil palm. This study aims to determine effects of auxin treatment and analyze differentially expressed genes in oil palm pistils at the pollination/anthesis stage, using an RNA sequencing (RNA seq) approach. The auxin treatment caused 100% parthenocarpy when auxin was sprayed before stigmas opened. The parthenocarpy decreased to 55%, 8% and 5% when the auxin was sprayed 1, 2 and 3 days after the opening of stigmas, respectively. Oil palm plants used for RNA seq were plants untreated with auxin as controls and auxin-treated plants on the day before pollination and 1 day after pollination. The number of raw reads ranged from 8,425,859 to 11,811,166 reads, with an average size ranging from 99 to 137 base pairs (bp). When compared with the oil palm transcriptome, the mapped reads ranged from 8,179,948 to 11,320,799 reads, representing 95.85–98.01% of the oil palm matching. Based on five comparisons between RNA seq of treatments and controls, and confirmation using reverse transcription polymerase chain reaction and quantitative real-time RT-PCR expression, five candidate genes, including probable indole-3-acetic acid (IAA)-amido synthetase GH3.8 (*EgGH3.8*), IAA-amido synthetase GH3.1 (*EgGH3.1*), IAA induced ARG7 like (*EgARG7*), tryptophan amino transferase-related protein 3-like (*EgTAA3*) and flavin-containing monooxygenase 1 (*EgFMO1*), were differentially expressed between auxin-treated and untreated samples. This evidence suggests a pathway of parthenocarpic fruit development at the beginning of fruit development. However, more research is needed to identify which genes are definitely involved in parthenocarpy.

## Introduction

Oil palm (*Elaeis guineensis* Jacq.) is a monoecious species that produces female and male inflorescences, in alternating cycles, on the same plant (Adam et al., 2011). Oil palm sex ratio is variable and affected by genetic and environmental factors. Female inflorescences are produced more in favorable conditions, such as adequate moisture, high light intensity and fertile soil whereas male inflorescences are produced in adverse conditions, such as low light intensity and water stress situations (Freeman et al., 1981; Williams & Thomas, 1970). In addition to sex determination, pollination and fertilization are also affected by adverse environmental conditions, such as light, temperature and humidity, resulting in failed fruit-set. However, parthenocarpic fruits, fleshy fruits without pollination or fertilization, have been produced under adverse environmental conditions (Klap et al., 2017; Martinelli et al., 2009; Pandolfini, 2009). In oil palm parthenocarpic fruit, oil-storing mesocarp is the major part of the fruit because of a lack of kernels or seeds. Improvement of parthenocarpic oil palm varieties can potentially increase the oil content in oil palm. In addition, parthenocarpic oil palm may produce parthenocarpic fruits in variable environments throughout the year. Therefore, basic knowledge of the parthenocarpy mechanism will help us to improve parthenocarpic oil palm for commercial use in the near future.

Natural parthenocarpy is caused by many factors, including natural mutations, environmental factors and hormonal regulators (Gustafson, 1938; Pandolfini, 2009). Gene regulation of oil palm parthenocarpic fruiting is partly understood. In 1973, Thomas et al. found that auxins were the predominate hormones that induce parthenocarpic fruits in oil palm but genetic control of parthenocarpy has been left unexplained.

In normal fertilized fruits, cross-talk signaling between fertilized ovules and growth of pistils was proposed to regulate fruit development after fertilization (Dorcey et al., 2009; Gillaspy, Ben-David & Gruissem, 1993). Unfertilized ovules resulted in undeveloped carpels and ovaries. However, carpels/pistils can develop into fruits without fertilized ovules by parthenocarpy, if the coordinated signaling between fertilized ovules and carpels/pistils is interrupted or modified naturally or artificially. Cross-talk interruption in fruit development and gene mutations contributing to parthenocarpy was reported most in tomatoes (Ficcadenti et al., 1999; Goetz et al., 2007; Ingrosso et al., 2011; Klap et al., 2017; Martí et al., 2007; Martinelli et al., 2009; Molesini et al., 2009; Serrani et al., 2007). These mutated genes are involved in development pathways mediated by growth/hormonal regulators, such as auxin, gibberillins (GAs), cytokinin and ethylene (Pandolfini, 2009). Interruption in auxin biosynthesis, signaling and response induces parthenocarpy. *Auxin Response Factor 8 (ARF8)* is known to act as an inhibitor for further carpel development in Arabidopsis in the absence of pollination/fertilization. Mutations in *ARF8* resulted in unfertilized, seedless fruit formation (Goetz et al., 2007, 2006). In auxin signaling, *ARF* regulated auxin-responsive genes, which were negatively controlled by *AUX/IAA* (Reed, 2001). However, in the presence of auxin, *AUX/IAA* is degraded by proteasomes and the expression of auxin-responsive genes is controlled by *ARF* transcription factors (Lau, Jürgens & De Smet, 2008). Down-regulation of *AUX/IAA* transcription factor (*IAA9*) induced parthenogenesis in tomato (Wang et al., 2005). The over-expression of genes in auxin biosynthesis may also induce parthenocarpy. For example, over-expression of an ovule-specific transgene, such as gene *iaaM*, which is an auxin synthase, induces obligated and facultative parthenocarpy (Martinelli et al., 2009). Dorcey et al. (2009) found that fertilization-dependent auxin response in ovules triggers fruit development through biosynthesis of GAs in Arabidopsis ovules and GAs signal ovary growth. However, another report has suggested that GAs are produced by pollen, which induces auxin production, which, in turn, signals for fruit-set and further fruit development (Gillaspy, Ben-David & Gruissem, 1993).

During early fruit development, high expression of GA biosynthetic enzyme genes, such as GA20ox and GA3ox, was found (Martí et al., 2007). Alteration of GA metabolism in mutants (*pat*, *pat2, pat3 and pat4*) developed tomato parthenocarpic fruits (Fos, Nuez & García-Martínez, 2000; Fos et al., 2001). GAs promote cell growth via degradation of DELLA proteins by proteasomes (Dill, Jung & Sun, 2001). Silencing of DELLA in GA signaling also induces facultative parthenocarpy in tomato fruits (Martí et al., 2007). Other evidence has shown that ethylene signaling can be exploited for seedless fruit. Overexpression of gene *SITPR1*, which interacts with ethylene receptors in ethylene signaling, also induces parthenocarpy in tomato fruit. Cross-talk between auxin and ethylene was suggested because the expression of auxin-responsive genes was inhibited by ethylene (Lin et al., 2008; Pandolfini, 2009; Wang et al., 2005).

Moreover, mutations in other pathways, such as the flavonoid biosynthesis pathway, has also been found to induce parthenocarpy. Schijlen et al. (2007) found that silencing chalcone synthase (CHS), an enzyme in the first step of the flavonoid biosynthesis pathway, induced parthenocarpy in tomato fruits. Silencing flavonol synthase, in the flavonoid biosynthesis pathway, also generated seedless tobacco fruits (Mahajan, Ahuja & Yadav, 2011). Overexpression of the stilbene synthase gene induced parthenocarpy and male sterility in tobacco and tomato (Fischer, Budde & Hain, 1997; Ingrosso et al., 2011). The relationship between flavonoids and parthenocarpy has never been clearly explained, except for the importance of flavonoids to pollen development and pollen tube growth (Schijlen et al., 2007; Ylstra et al., 1992). However, loss of CHS activity in Arabidopsis caused an increase in polar auxin transport and it was proposed that flavonoids act as endogenous negative regulators for auxin transport (Wasson, Pellerone & Mathesius, 2006). Changes in endogenous flavonoid concentration led to changes in auxin transport. Moreover, flavonoid biosynthesis was found to be highly regulated by environmental factors, such as light, wounding, pathogens and symbiotic bacteria (Sakuta, 2000; Schmid et al., 1990). For example, CHS is induced by UV and blue light (Feinbaum & Ausubel, 1988; Feinbaum, Storz & Ausubel, 1991; Jackson et al., 1995). Parthenocarpy results from an altered distribution of auxin, which is caused by reduced levels of flavonoids. Clearly, fruit development is a complex process involving many plant growth regulators and cross-talk between plant hormones.

This study aims to first determine the effect of auxin treatment on oil palm parthenocarpy at the anthesis stage. We analyzed differentially expressed genes (DEGs) in oil palm pistils at the pollination/anthesis stage, using RNA seq-based comparisons between oil palm female inflorescences treated with auxin and the control inflorescences without auxin treatment. Subsequently, the promising candidates were selected and their expression was confirmed via reverse transcription polymerase chain reaction (RT-PCR) and quantitative real-time RT-PCR (qRT-PCR).

## Materials and Methods

### Plant materials

An oil palm variety, commercially named as Surat Thani 2, was selected for our study because of its long bunch stalks, allowing for clear female flower visibility. A total of 15 6-year-old oil palm plants with visible female inflorescences during an anthesis stage were selected. Oil palm, Surat Thani 2, is a cross between Deli dura (mother plant) and Lame (father plant), developed by Department of Agriculture, Ministry of Agriculture and Cooperatives, Thailand (http://www.doa.go.th/main/). The oil palm trees were grown in Saraburi, Thailand by the Oil Palm Technology Development for commercial bio diesel industry in newly planted area project (OPTD), Department of Agronomy, Faculty of Agriculture, Kasetsart University (Bangken Campus).

### Hormonal treatment and collection of oil palm pistils

Synthetic auxin, 2, 4, 5-tri-chlorophenoxy propionic acid (2, 4, 5-TP), which has been reported to induce parthenocarpy in oil palm (Thomas et al., 1973), was used for spraying the female inflorescences. One gram of 2, 4, 5-TP was first dissolved in 100 ml methanol. The solution was poured into a five l pressure sprayer and then filled with distilled water to make a total volume of five l. About 250 ml (about 50 mg of 2, 4, 5-TP) of the solution was sprayed on each inflorescence, using the pressure sprayer. The treatments were performed on three consecutive days during different pollination stages and again after 7 and 14 days. The oil palm plants with visible female inflorescences selected for the treatment had female inflorescences in different stages of anthesis. These anthesis stages included the stage with unopened stigma, assigned as day after pollination (DAP) = 0, stigma opened for 1 day (DAP = 1), stigma opened for 2 days (DAP = 2) and 3 days (DAP = 3). So, the stages with the first day of 2, 4, 5-TP spraying included DAP = 0, DAP = 1, DAP = 2 and DAP = 3. In summary, 13 female inflorescences (named as Inflo. 1T-13T) of 13 oil palm trees of four different pollination stages were subjected to auxin treatment and four female inflorescences (Inflo. 1C-4C) of four oil palm trees were used as the controls, without auxin treatment. Details of the oil palm trees used for both treated and untreated inflorescences and the first day of treated stages are listed in Table S1. The parthenocarpy phenotype was observed 2 weeks, 6 weeks and at the ripening stage (18 weeks) after pollination. For the auxin treated oil palm plants, five female flowers were collected for each inflorescence before the first auxin spraying and again 24 and 48 h after the spraying, for RNA extraction. The collected flowers were immediately frozen in liquid nitrogen during collection and stored at −80 °C until the extraction was performed. For the control oil palm plants, without the auxin treatment, the pistil samples were collected at the same stages as were the treatments.

### Summary of RNA samples and pooling strategy

For expression studies, oil palm plants that were treated with auxin on DAP = 0 and DAP = 1 were selected because spraying during these stages appeared to result in a higher degree of parthenocarpy. Details of the three control and three auxin-treated inflorescences, used for pistil collection at the given stages, are represented on Table S2. The RNA samples used as the auxin treated group included Inflo.1T/WA, Inflo.6T/WA and Inflo.4T/WA. The RNA samples used as the control group included Inflo.1T/NA, Inflo.1C/NA, Inflo.2C/NA1, Inflo.2C/NA2 and Inflo.4C/NA. Since the total RNA for each pollination stage of some samples was not enough for mRNA isolation, some RNA samples were pooled. The pooled samples included RNA samples, Inflo.1T/WA (24 and 48 h auxin-treated samples were pooled), Inflo.6T/WA (24 and 48 h auxin-treated samples were pooled), Inflo.4T/WA (24 and 48 h auxin-treated samples were pooled), Inflo.1C/NA (samples that were not auxin-treated at DAP = 0 of day 2 and 3 collection were pooled), Inflo.2C/NA1 (samples that were not auxin-treated at DAP = 1 and 2 were pooled) and Inflo.4C/NA (samples that were not auxin-treated at DAP = 2 and 3 were pooled) (Table S2).

### RNA extraction, mRNA isolation and mRNA purification

RNA extraction was performed on a large scale by the CTAB method. A total RNA of about 75 μg was needed for mRNA isolation. Five oil palm pistils for each inflorescence were ground in liquid nitrogen and transferred to five two ml tubes containing 1,000 μl extraction buffer (2% CTAB, 1.4 M NaCl, 2% PVP, 20 mM EDTA pH 8, 100 mM Tris–HCl pH 8) and 20 μl of 20% SDS. After adding β-mercaptoethanol, the extraction was cleaned with chloroform: Isoamyl (24:1) for five times. The final supernatant was precipitated with 1/3 volume of 8M LiCl resulting in a final concentration of 2M LiCl. The pellet was washed with 70% ethanol, then air dried and resuspended in 50–100 μl of DEPC water. The quality and concentration was determined by 1% agarose gel electrophoresis and NanoDrop^®^ND-1000.

Isolation of mRNA was performed using the Dynabeads mRNA purification kit (Thermo Fisher Scientific Baltics UAB, Vilnius, Lithuania). About 75 μg of total RNA was needed and dissolved in DEPC water to make final volume of 100 μl. If the RNA sample was less than 75 μg diluted in more than 100 μl of solution, whole volume was used. After purification by the kit, the mRNA was eluted by adding 25 μl of 10 mM Tris–HCl pH 7.5. The quality and concentration were determined on a Fragment Analyzer (Advanced Analytical Technologies, Inc., Ames, IA, USA), using the DNF-472M33 kit. The mRNA was diluted to about 1,000–2,500 pg/μl and two μl of mRNA was used for the determination. Purification of mRNA was performed by using the Magnetic Bead Cleanup Module (Thermo Fisher Scientific, Waltham, MA, USA). Five μl of 37 °C pre-warmed nuclease-free water was used to elute purified mRNA and the solution was kept for preparing the barcoded RNA library.

### Barcoded RNA library preparation

The barcoded RNA libraries were made following the Ion Total RNA-Seq Kit v2 (Life Technologies, Carlsbad, CA, USA) protocol. Briefly, all five μl of mRNA from the previous step was hybridized and ligated with the Ion Adaptor Mix v2. The ligation reaction was performed on a Veriti 96 well Thermal Cycler (Applied Biosystems, Foster City, CA, USA), at 30 °C for 1 h. Then, reverse transcription was performed using SuperScript^®^ III Enzyme Mix and incubated at 42 °C for 30 min. The cDNA was purified by a magnetic bead cleanup module. A total of 12 μl of 37 °C pre-warmed nuclease-free water was used to elute the purified cDNA. Next, the barcodes were added in the amplifying cDNA step. Six μl of purified cDNA was used. The barcoded libraries were made using Platinum^®^ PCR SuperMix High Fidelity, Ion Xpress™ RNA 3′ Barcode primer and Ion Xpress™ RNA-Seq Barcode BC primer. In this study, the BC primers included BC1-BC8 and BC12. The reaction was run on a thermal cycler with the following setting. The reaction was first incubated at 94 °C for 2 min, then performed for two cycles at 94 °C for 30 s, 50 °C for 30 s, 68 °C for 30 s, then performed for 16 cycles at 94 °C for 30 s, 62 °C for 30 s, 68 °C for 30 s and ended at 68 °C for 5 min. The amplified cDNA was also purified using a magnetic bead cleanup. A total of 15 μl of 37 °C pre-warmed nuclease-free water was used to elute the purified amplified cDNA.

The quality and concentration were determined on a Fragment Analyzer (Advanced Analytical Technologies, Inc., Ames, IA, USA), using the DNF-474-33 kit (HS NGS fragment 1-6000 bp). The cDNA was diluted to about 2,500–5,000 pg/μl and two μl of cDNA was also used for the determination.

### Ion proton sequencing and transcriptional analysis

Details of RNA sequencing are explained in the Ion 540™ Kit-OT2 User guide. Briefly, each barcoded cDNA library from the previous step was diluted to 100 pM. Then, the libraries were pooled and six μl of the pooled cDNA was used for template preparation. The template-positive Ion 540™ ion sphere particles (ISPs), containing amplified cDNA (about 200 bp), were prepared using the Ion 540™ kit-OT2 kit and the reaction was performed in the One Tough™ 2 Instrument. The template-positive ISPs were enriched using Ion One Tough™ ES. Next, the enriched template-positive ISPs were loaded on an Ion 540™ Chip and sequenced on an Ion S5™ XL sequencer.

For the expression analysis, raw reads were first mapped to the oil palm genome (https://www.ncbi.nlm.nih.gov/genome/2669), which is *E. guineensis* assembly EG5, by using bowtie2-2.2.3. Reads in gene regions were counted by HT-seq count version 0.9.1 .The raw read count was normalized using the R package DESeq2. The expression fold-change of each gene was calculated between treatment and control conditions with the threshold for DEGs set to *p*-value < 0.05.

### Reverse transcription polymerase chain reaction analysis and quantitative real-time RT-PCR of candidate genes controlling parthenocarpy

To confirm the RNA seq expression of candidate genes, the same amplified cDNA samples of mRNA used for RNA seq were subjected to RT-PCR. The RT-PCR reaction was performed in three replicates, for both target and reference genes. Cyclophilin 2 (renamed as *EgCyp2*) was chosen for the reference gene (Yeap et al., 2014). The primer sequences were 5′ CTCGTCTGATGTCGTCTA 3′ as the forward primer and 5′ CTGCTGGTACTCTGGTAA 3′ as the reverse primer. The RT-PCR reaction was conducted in a 20 μl solution, containing three μl of cDNA (one ng/μl of the amplified cDNA), 1× High Fidelity buffer, two mM MgSO_4_, 400 μM dNTP, 0.5 μM of each primer and 0.5 unit of Platinum^®^Tag DNA polymerase High Fidelity (Invitrogen by Thermo Fisher Scientific, Waltham, MA, USA). The amplification was performed on the Veriti 96 Well Thermocycler (Applied Biosystems by Thermo Fisher Scientific, Waltham, MA, USA). The PCR reaction was set as the following; 5 min at 94 °C of initiation PCR activation, 35 cycles of 1 min at 94 °C, 0.30 min at 55 °C and 1 min at 68 °C. The reaction ended in a 7 min-step at 68 °C. The products were analyzed on 1% agarose gel, containing Invitrogen SYBR safe DNA gel stain.

Quantitative real-time RT-PCR was performed using Fast Start SYBR Green Master (Roche Applied Science, Penzberg, Germany). The same cDNA and reference gene, *EgCyp2*, as RT-PCR was used in this qRT-PCR. The reaction was conducted in a 20 μl solution, containing three μl of cDNA (one ng/μl of the amplified cDNA), 1× FastStart SYBR Green Master, 0.5 μM of each primer. The qRT-PCR reaction was performed in three replicates, for both target and reference genes. The amplification was performed on the CFX 96 Real-Time PCR detection system (Bio-Rad Laboratories, Inc., Hercules, CA, USA) at this setting; one cycle of 3 min at 95 °C, 50 cycles of 0.10 min at 95 °C, 0.15 min at 55 °C and 0.30 min at 72 °C, then the reaction was ended with 95 °C for 0.10 min. This was followed with the melting steps from 65 °C to 95 °C by increments of 0.5 °C for 0.05 min in each step. The fold expression was calculated using the normalized expression (ΔΔCq) method, normalized to *EgCyp2* expression by CFX manager software. Settings of the graph data were *X*-axis as target, *Y*-axis as linear, scaling as unscaled and error type as standard error of the mean.

## Results

### Characteristics of stigmas during different pollination stages

The characteristics of stigma during pollination indicate the occurrence of pollen-pistil interaction. If a stigma is still white and closed, it means that pollen-pistil interaction has not occurred. We assigned stage DAP = 0 as the stage in which the stigma is still white and closed and pollination has not taken place. The pollination usually occurs during DAP = 1 to DAP = 2 stages because the white stigma is still opened for day 1 and day 2, respectively. The stigma has a light pink color during stage DAP = 3, a dark pink color during DAP = 4, and then a dry black color by DAP = 10. Auxin treatment may be most effective for inducing parthenocarpy if pollination has not occurred. This work started spraying auxin in four different stages, DAP = 0, DAP = 1, DAP = 2 and DAP = 3, as shown in Fig. S1.

### The effect of auxin treatment on parthenocarpy before the ripening stage

Before the ripening stage, the effect of auxin on parthenocarpy was observed 1, 2 and 6 weeks after pollination. Several characteristics were recorded, including stigma characteristics, such as color, opening or closing, dryness, the fertility of fruits, fruit shape and the presence of kernels. In control inflorescences, normal fruits developed 6 weeks after pollination. The fruits contained soft shells and liquid endosperm, which is part of the kernel. Black dry stigmas were still attached at the top of the fruits. However, there were several changes found in the treated fruits, when compared with the control fruits. Stigmas of the treated fruits (first treated at DAP =1, DAP = 2 and DAP = 3) were still fresh and red for 2 weeks while stigmas of the control fruits were dry and black since DAP = 5, as in Fig. S2A. Moreover, stigmas of Inflo.1T, first treated with auxin at DAP = 0, were still fresh and red, and the freshness and redness lasted for 6 weeks after pollination, as shown in Fig. S2B. The development of treated oil palm fruits of Inflo.4T was delayed, causing fruits to be smaller than those of controls. Some fruits, including some from Inflo.5T and Inflo.8T, failed to develop. The shape of fruits produced by treated plants was more oval than that of the controls, which produced more rounded fruits. However, the majority of treated oil palm fruits developed to the ripening stage successfully. In addition, auxin had an effect on oil palm plants that were treated on DAP = 3 because the fruits developed kernels. The development, however, was slower than that of the controls.

### The effect of auxin treatment on parthenocarpy at the ripening stage

**Figure 1:**
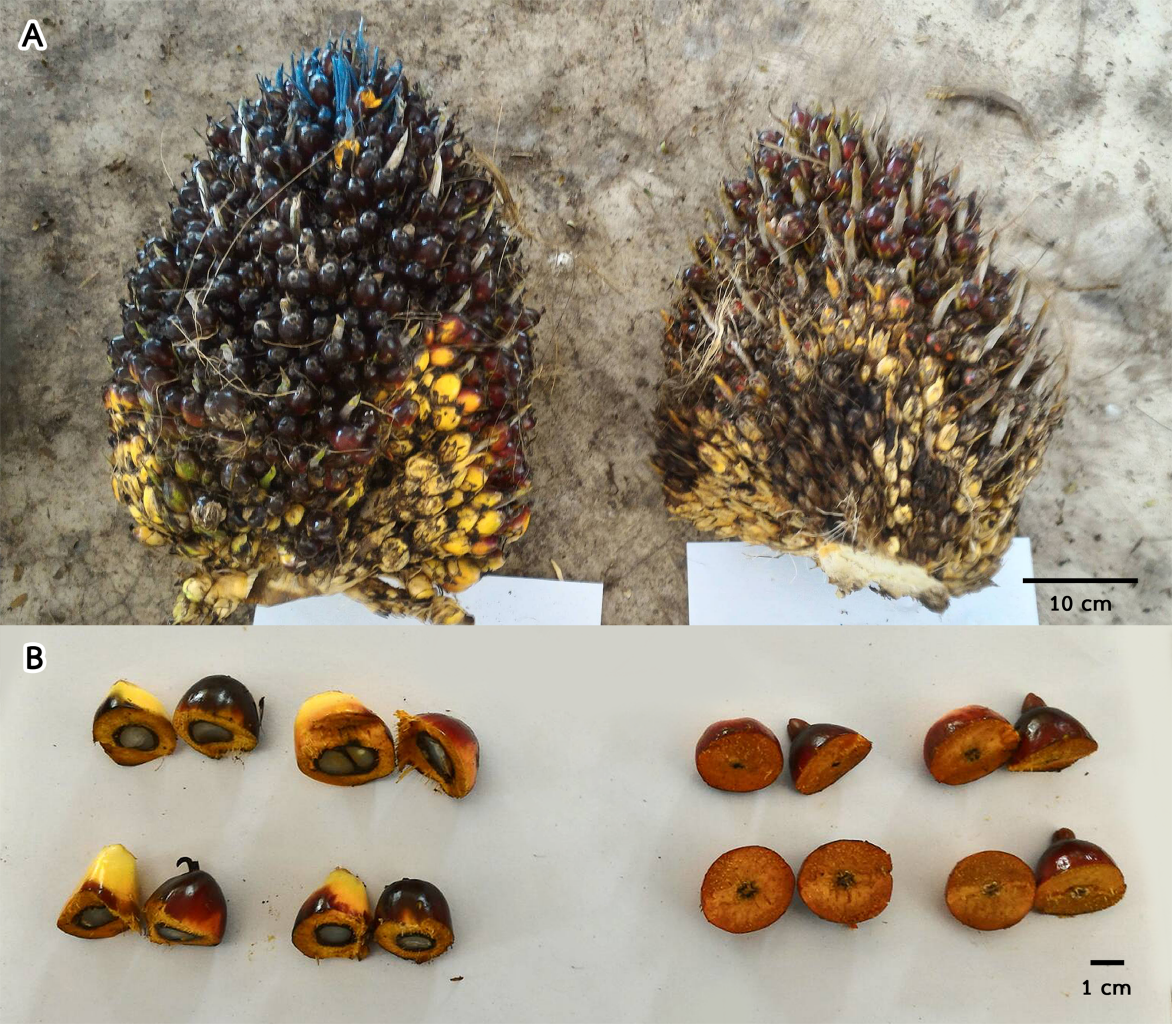
A comparison of oil palm bunches (A) and cross sections of fruits (B) between inflo.1C control fruits (left) and inflo.1T treated fruits (right) showing that the auxin treated fruits displayed 100% parthenocarpy. cm = size indicator in centimeter.

**Figure 2:**
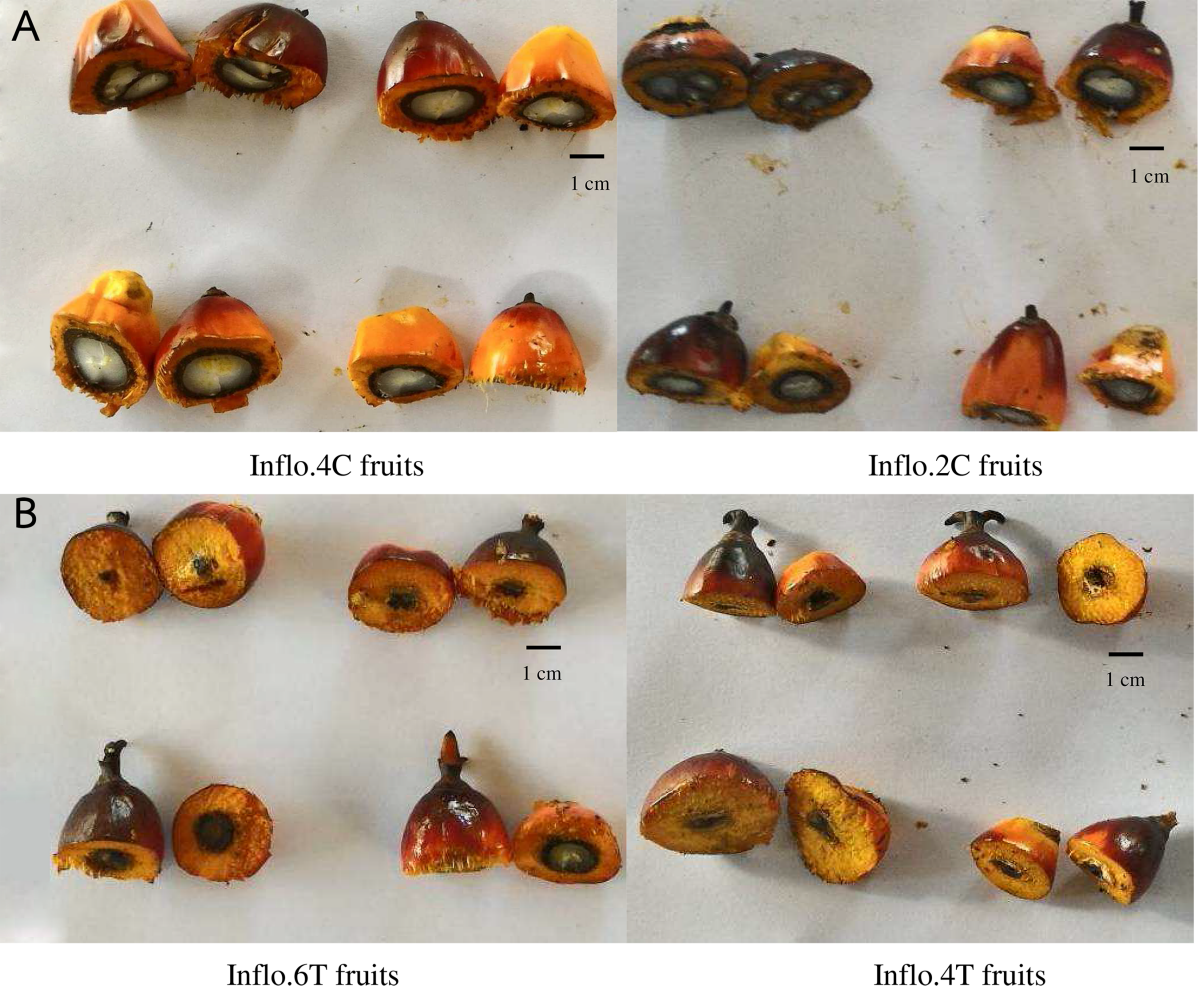
A comparison of oil palm fruit cross sections between the control fruits of Inflo.4C and Inflo.2C (A), without auxin treatment, and the auxin treated fruits of Inflo.6T and Inflo.4T, which had 50% and 90% parthenocarpy, respectively (B). The auxin treatment was first sprayed on DAP = 1 of anthesis. cm = size indicator in centimeter.

The effect of auxin on parthenocarpy was observed about 4.5 months after pollination (ripening stage) that seedless fruits was the most visible by cutting fruits in half. All fruits of the control oil palm plants (Inflo.1C-4C) contained kernels, hard shells and oily mesocarp. Auxin treatment had the greatest parthenocarpic effect when auxin was sprayed on DAP = 0, when stigmas had not yet opened. This experiment was performed on two oil palm trees (Inflo.1T and Inflo.2T). The treated bunches were smaller than the controls (Fig. 1A) and the treated fruits showed 100% parthenocarpy (*n* = 30), without seeds (no white kernel), as shown in Fig. 1B. When auxin was first sprayed on DAP = 1, the effect of auxin on parthenocarpy was reduced because some pistils were pollinated. The experiment was conducted on four oil palm plants (Inflo.3T-Inflo.6T). The fruit set of Inflo.5T failed, while the other three oil palms showed varying degrees of parthenocarpy, with Inflo.3T, Inflo.6T and Inflo.4T having 27%, 50% and 90% parthenocarpic fruits from 20 randomly selected fruits for each oil palm plant, as shown in Fig. 2. Moreover, the effect of auxin on parthenocarpy was reduced further on DAP = 2 because pistils were pollinated more 2 days after stigma opening. This auxin treatment was performed on DAP = 2 in four oil palm trees (Inflo.7T-Inflo.10T). The levels of parthenocarpy were variable with Inflo.10T, Inflo.7T, Inflo.8T and Inflo.9T having 5%, 6%, and 10% parthenocarpic fruits from 20 randomly selected fruits for each oil palm, respectively. Even though the pollen-pistil interaction was expected to stop at DAP = 3, we still found that auxin treatment induced some parthenocarpic fruits but to a smaller degree. These oil palms including Inflo.12T, Inflo.11T and Inflo.13T, which contained 0%, 5% and 10% parthenocarpic fruits from 20 randomly selected fruits for each oil palm, respectively. The above information indicates that hormone treatment can induce parthenocarpic development throughout fruit progression to the ripening stage. The hormonal treatment caused 100%, 55%, 8% and 5% parthenocarpy when the inflorescences were auxin-treated on DAP = 0, DAP = 1, DAP = 2 and DAP = 3, respectively.

### RNA sequencing and data analysis

Eight barcoded RNA libraries from the above eight RNA samples were used for Ion Proton sequencing. Raw reads ranged from 8,425,859 to 11,811,166 reads, with average sizes ranging from 99 to 137 bp, as shown in Table 1. The raw data was deposited in the NCBI database (https://www.ncbi.nlm.nih.gov/) (SRA accession: PRJNA490783). After comparing with the reference oil palm genome (assembly EG5), the mapped reads ranged from 8,179,948 to 11,320,799 reads, representing 95.85–98.01% of the map. The DEGs of the auxin-treated oil palms were compared with those of the control oil palms. There are two groups that were separated for comparisons. For oil palms that were first treated on DAP = 0, the data for comparison includes RNA expression data from RNA libraries, Inflo.1T/NA, Inflo.1T/WA and Inflo.1C/NA. For oil palms that were first treated on DAP = 1, the data for comparison includes RNA expression data from RNA libraries, Inflo.6T/WA, Inflo.4T/WA, Inflo.2C/NA1, Inflo.2C/NA2 and Inflo.4C/NA. The expression-fold-change of each gene was calculated between treatment and control oil palm plants with the threshold for DEGs set to a *p*-value <0.05. To reveal DEGs, eight pair comparisons were performed between treated and control samples, listed in Table S3. The number of DEGs found was from 120 to 246 genes, for each library. About 9% of the DEGs are categorized by KEGG (Kyoto Encyclopedia of Genes and Genomes; http://www.genome.jp/kegg/) (Fig. S3). A total of 83 from a total of 136 KEGG categorized DEGs are nonredundant genes. The KEGG pathway that contains the highest number of down-regulated DEGs is amino sugar and nucleotide sugar metabolism. These genes included chitinase 1-like, endochitinase EP3-like, GDP-mannose dehydratase 1-like, and UDP-glucose 6-dehydrogenase 5-like. The KEGG pathway that contains the most up-regulated DEGs is drug metabolisms, including cytochrome P450, metabolism of xenobiotics by cytochrome P450 and glutathione metabolism. These genes included glutathione S-transferase U8-like, probable carboxylesterase 18 and probable glutathione S-transferase GSTF1. Some DEGs that are not categorized by KEGG are in the auxin signaling pathway, which is the pathway that we are focusing on in this experiment. These genes are the potential candidate genes involved in parthenocarpy in oil palm.

**Table 1:** Details of raw reads of RNA seq from Ion Proton and percentage of sequence matching with oil palm reference sequences.

Barcoded library names	Bases	> = Q20 Bases	Reads	Mean read length (bp)	Mapped read	% Mappings
Inflo.1T/NA	1,099,812,072	1,001,462,598	8,828,407	124	8554344	96.9
Inflo.1T/WA	1,169,781,850	1,081,165,713	11,811,166	99	11320799	95.85
Inflo.1C/NA	1,221,657,984	1,108,089,612	8,880,263	137	8703949	98.01
Inflo.6T/WA	1,074,037,592	981,486,821	9,002,657	119	8648968	96.07
Inflo.4T/WA	1,124,732,016	1,025,042,567	8,425,859	133	8179948	97.08
Inflo.2C/NA1	1,216,787,891	1,107,754,438	9,607,194	126	9348674	97.31
Inflo.2C/NA2	1,207,249,741	1,105,974,168	10,764,142	112	10334242	96.01
Inflo.4C/NA	1,403,686,167	1,282,011,864	11,597,849	121	11118594	95.87

### Candidate genes in the auxin signaling pathway controlling parthenocarpy

We proposed candidate genes involved in the auxin signaling pathway by the eight pair comparisons explained earlier. Five out of eight comparisons between the treatments and controls revealed six candidate genes in the auxin signaling pathway, showing differential expression during auxin treatment on DAP = 0 and DAP = 1, as shown in Table 2. In the treatment at DAP = 0 group, two candidate genes were found, including probable flavin-containing monooxygenase 1 (cds26665) and auxin-responsive protein SAUR71-like (cds32638). In the treatment at DAP = 1 group, four candidate genes were found, including probable indole-3-acetic acid (IAA)-amido synthetase GH3.8 (cds27914), probable IAA-amido synthetase GH3.1 (cds30335), IAA-induced protein ARG7-like (cds31450) and tryptophan aminotransferase-related protein 3-like (cds7351).

**Table 2:** Details of the differentially expressed candidate genes (DEGs) in the auxin signaling pathway, found during the auxin treatment.

Treatments	Controls	Genes	log2FoldChange	Padj	Contig	Product	Assigned gene name
Inflo.1T/WA	Inflo.1C/NA	cds26665	−2.56453527	4.84E-05	NC_026004.1	Probable flavin-containing monooxygenase 1	*EgFMO1*
		cds32638	−3.52607636	0.01279948	NW_011550905.1	Auxin-responsive protein SAUR71-like	*EgSAUR71*
Inflo.1T/WA	Inflo.1T/NA	cds26665	−1.72066514	0.00607568	NC_026004.1	Probable flavin-containing monooxygenase 1	*EgFMO1*
Inflo.6T/WA	Inflo.2C/NA2	cds27914	5.623534449	0.003077281	NC_026005.1	Probable indole-3-acetic acid-amido synthetase GH3.8	*EgGH3.8*
		cds30335	5.250355765	0.028257725	NC_026007.1	Probable indole-3-acetic acid-amido synthetase GH3.1	*EgGH3.1*
Inflo.6T/WA	Inflo.2C/NA1	cds27914	6.204689246	0.001123803	NC_026005.1	Probable indole-3-acetic acid-amido synthetase GH3.8	*EgGH3.8*
		cds30335	5.828605964	0.014649213	NC_026007.1	Probable indole-3-acetic acid-amido synthetase GH3.1	*EgGH3.1*
		cds31450	−6.914624815	0.007448309	NC_026008.1	indole-3-acetic acid-induced protein ARG7-like	*EgARG7*
Inflo.6T/WA	Inflo.4C/NA	cds27914	2.00227083	0.02941347	NC_026005.1	Probable indole-3-acetic acid-amido synthetase GH3.8	*EgGH3.8*
		cds7351	−4.56093586	0.01115662	NC_025995.1	Tryptophan aminotransferase-related protein 3-like	*EgTAA3*

**Note:**

Log2FoldChage > 0 means that DEGs were up-regulated in the treatments while Log2FoldChage < 0 means that DEGs were down-regulated in the treatments, compared with controls.

From plants treated with auxin on DAP = 0, we found flavin-containing monooxygenase 1 (*EgFMO1*) to be the strongest candidate gene for parthenocarpy because it showed differential expression, when compared with the two controls, Inflo.1T/NA and Inflo.1C/NA. Another gene, auxin-responsive protein SAUR71-like (*EgSAUR71*) was found to be differentially expressed, compared to Inflo.1C/NA. In plants treated on DAP = 1, probable IAA-amido synthetase GH3.8 (*EgGH3.8*) is the strongest candidate for parthenocarpy because it was differentially expressed, when compared with the three controls, Inflo.2C/NA1, Inflo.2C/NA2 and Inflo.4C/NA. The next potential candidate for parthenocarpy is probable IAA-amido synthetase GH3.1 (*EgGH3.1*) because it was differentially expressed, compared to the two controls, Inflo.2C/NA1 and Inflo.2C/NA2. IAA-induced protein ARG7-like (*EgARG7*) and tryptophan aminotransferase-related protein 3-like (*EgTAA3*) were differentially expressed compared with one control. Inflo.2C/NA1 and Inflo.4C/NA, respectively.

### Expression confirmation of the candidate genes in the auxin signaling pathway by reverse transcription polymerase chain reaction and quantitative real-time RT-PCR

**Figure 3:**
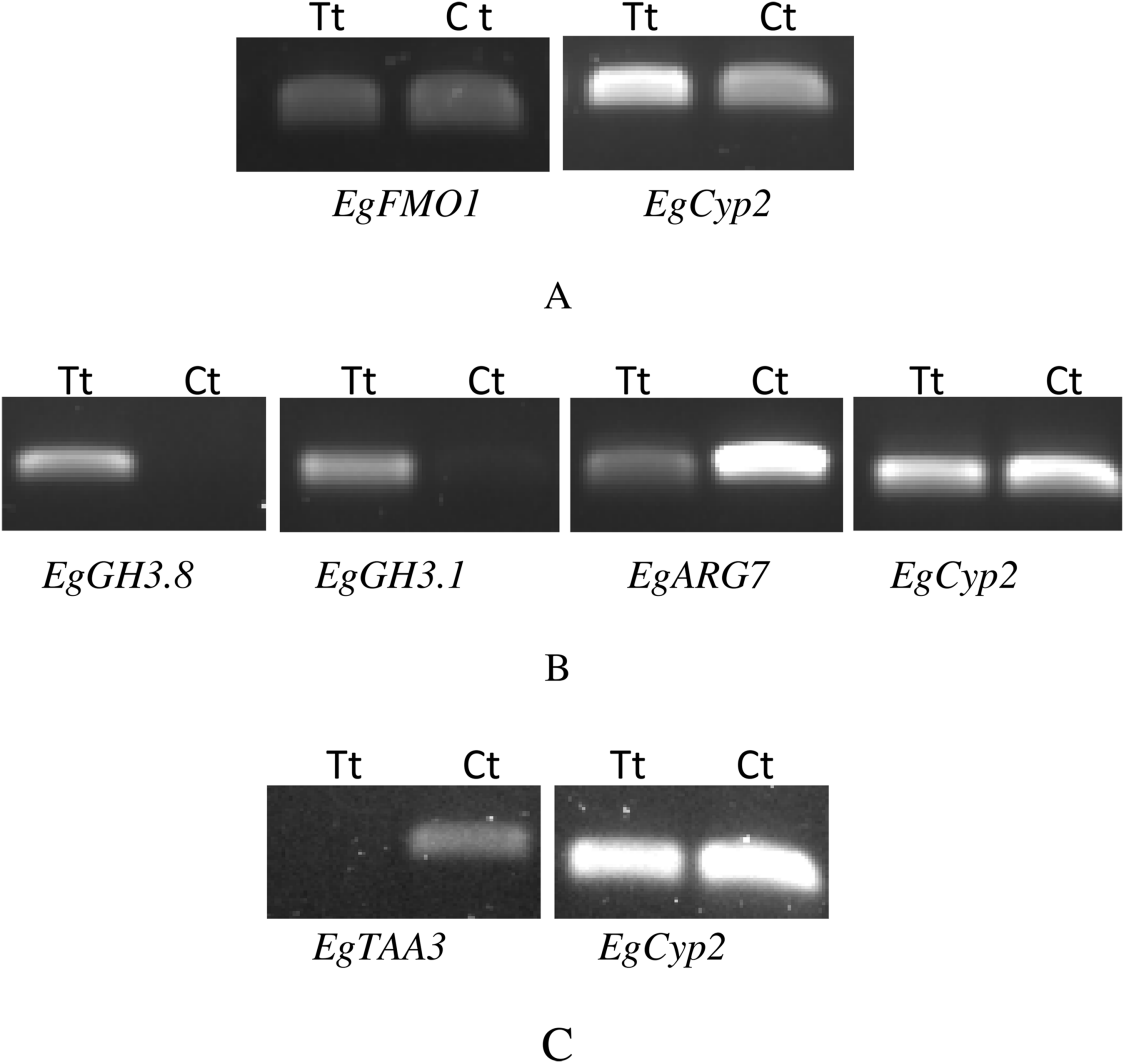
RT-PCR of candidate genes that were expressed differentially between auxin treated samples (Tt) and the control samples (Ct). The first RT-PCR comparison was between Inflo.1T/WA (Tt) and Inflo.1C/NA (Ct), by searching for differences in *EgFMO1* amplification (A). The second RT-PCR comparison was between Inflo.6T/WA (Tt) and Inflo.2C/NA1 (Ct), by searching for differences in *EgGH3.8*, *EgGH3.1* and *EgARG7* amplification (B). The third RT-PCR comparison was between Inflo.6T/WA (Tt) and inflo.4C/NA (Ct), by searching for differences in *EgTAA3* amplification (C). *EgCyp2* was used as the reference gene in all three comparisons.

**Figure 4:**
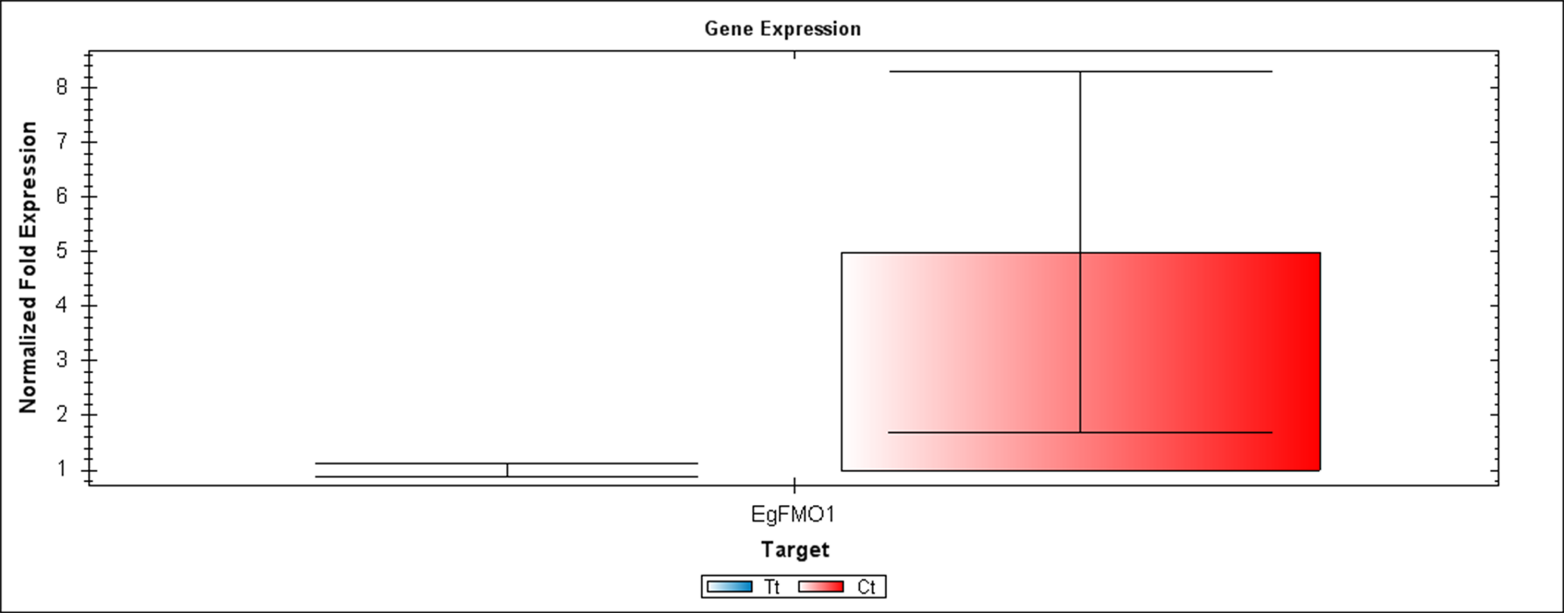
A qRT-PCR comparison of *EgFMO1* expression between Inflo.1T/WA (Tt) and Inflo.1C/NA (Ct). *EgCyp2* was used as the reference gene. Tt = an auxin treated sample, Ct = a control sample without auxin treatment.

**Figure 5:**
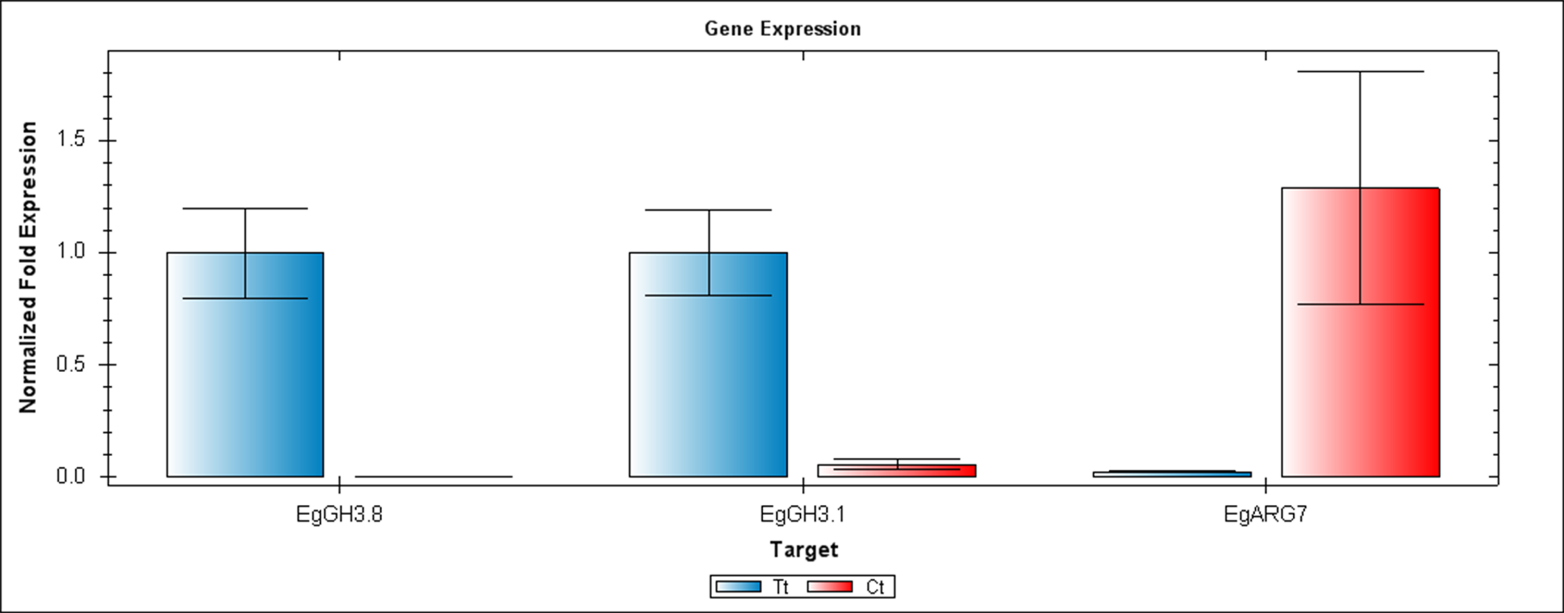
QRT-PCR comparisons of *EgGH3.8*, *EgFH3.1* and *EgARG7* expression between Inflo.6T/WA (Tt) and Inflo.2C/NA1 (Ct). *EgCyp2* was used as the reference gene. Tt = an auxin treated sample, Ct = a control sample without auxin treatment.

**Figure 6:**
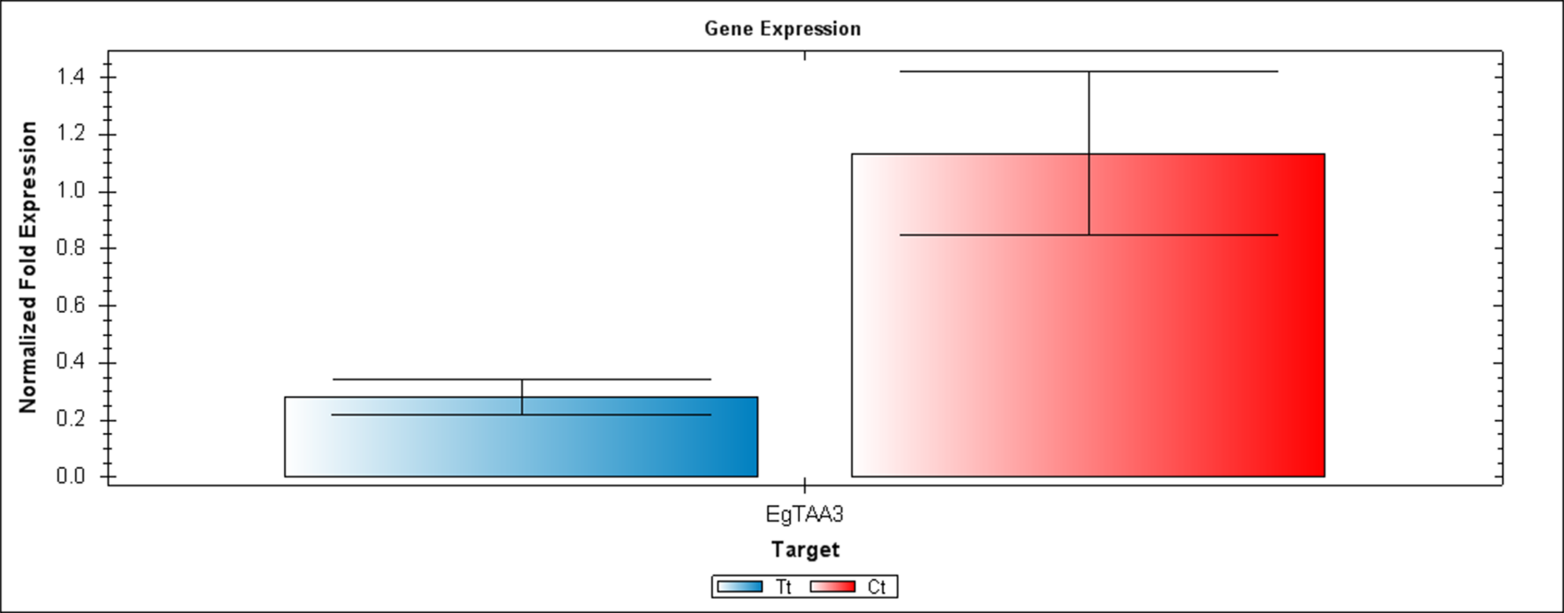
A qRT-PCR comparison of *EgTAA3* expression between Inflo.6T/WA (Tt) and inflo.4C/NA (Ct). *EgCyp2* was used as the reference gene. Tt = an auxin treated sample, Ct = a control sample without auxin treatment.

We performed RT-PCR and qRT-PCR experiments confirming RNA seq results by focusing on the auxin signaling genes. The sequences of the six candidate genes that were used to design primers for RT-PCR and qRT-PCR are available on the NCBI database. The accession numbers and details of primers that were used to amplify these six genes are listed in Table S4. Confirming by RT-PCR and qRT-PCR, five candidate genes were found to be clearly differentially expressed between treated and control samples, as shown in Figs. 3–6. *EgCyp2* was used as the reference gene with an expected size of 163 bp. These candidate genes included probable IAA-amido synthetase GH3.8 (*EgGH3.8*), IAA-amido synthetase GH3.1 (*EgGH3.1*), IAA induced ARG7 like (*EgARG7*), tryptophan amino transferase-related protein 3-like (*EgTAA3*) and flavin-containing monooxygenase 1 (*EgFMO1*).

The first RT-PCR comparison was between Inflo.1T/WA and Inflo.1C/NA, by searching for differences in *EgFMO1 and EgSAUR71* amplifications. The *EgFMO1* expression was not clearly different between the treatment and controls (Fig. 3A). The amplification of *EgSAUR71* by the two given primers (Table S4) failed. However, after confirming by qRT-PCR, the *EgFMO1* expression was found more in the control than it was in the treatment, as shown in Fig. 4. This result correlates with RNA seq that shows expression of *EgFMO1* in the control to be about 2.5 times greater than it is in the treatment. The second RT-PCR and qRT-PCR comparisons were between Inflo.6T/WA and Inflo.2C/NA1, by searching for differences in *EgGH3.8*, *EgGH3.1* and *EgARG7* amplifications (Figs. 3B and 5). The *EgGH3.8* expression was only found in the treatment. This result correlates with RNA seq that shows expression of *EgGH3.8* in the treatment to be about 6.2 times greater than it is in the control. *EgGH3.1* expression was also only found in the treatment. This result also correlates with RNA seq that shows expression of *EgGH3.1* in the treatment to be about 5.8 times greater than it is in the control. *EgARG7* expression in the control was more than in the treatment. This result correlates with RNA seq that shows expression of *EgARG7* in the control to be about 6.9 times greater than in the treatment. The third RT-PCR and qRT-PCR comparisons were between Inflo.6T/WA and inflo.4C/NA, by searching for differences in *EgTAA3* amplification (Figs. 3C and 6). The *EgTAA3* expression was found more in the control than it was in the treatment. This result correlates with RNA seq that shows expression of *EgTAA3* in the control to be about 4.6 times greater than in the treatment. *EgCyp2* was used as the reference gene in all three comparisons.

## Discussion

Synthetic auxin, 2, 4, 5-TP was chosen to induce parthenocarpy because it has been reported to induce complete parthenocarpy (Thomas et al., 1973). Other hormones, including NAA, 2 CPA, 2,4-D, 2,4,5-T, IBA, GA_3_, 4 CPA and IPA, were effective in varying degrees in decreased range order, respectively. The treatment was performed on commercial oil palm, named as Surat Thani 2 because of its long bunch stalk, causing high female flower visibility. Auxin is the predominant hormone inducing parthenocarpy in many plant species, including Arabidopsis, tomatoes and eggplant (Acciarri et al., 2002; Ficcadenti et al., 1999; Goetz et al., 2007; Gorguet et al., 2008; Ingrosso et al., 2011; Klap et al., 2017; Martí et al., 2007; Martinelli et al., 2009; Molesini et al., 2009; Serrani et al., 2007; Wang et al., 2005). GAs, Ethylene and cytokinin were also reported to induce parthenocarpy in tomatoes, and cucumber (Ding et al., 2013; Fos, Nuez & García-Martínez, 2000; Lin et al., 2008; Martí et al., 2007; Pandolfini, 2009; Wang et al., 2005). Clearly, fruit development is a complex process involving many plant growth regulators and cross-talk between plant hormones. In this work, we try to examine the effect of auxin spraying in four different stages, DAP = 0, DAP = 1, DAP = 2 and DAP = 3. The first change observed was a delay in stigma decay. When flowers were sprayed before stigmas opened, the stigmas stayed closed and kept their fresh red color for more than 2 weeks. When flowers were sprayed at the time when stigmas were opening, they continued to open but kept their red color and dried out slower than the control group stigmas.

To manipulate parthenocarpy in oil palm, its pollination, fertilization and pollen-pistil interactions should be understood. Anthesis to pollination takes 24 h. Tandon et al. (2001) reported that about 15–26% of the total falling pollen germinates on stigmatic lobes, 10–14 h before stigmas completely opens. Most pollen (about 67–76%) germinates on stigmatic lobes which are fully opened. A total of 0–13% of pollen germination was found after 24 h of anthesis. After 24 h of anthesis, anthocyanin is developed, indicating a loss of receptivity. In our study, the hormonal treatment causing parthenocarpy seems to correspond to the number of non-fertilized female flowers. The hormonal treatment causes complete parthenocarpy when auxin treatment is performed on unopened female flowers. When the stigmatic lobes were fully opened, the hormonal treatment caused some degree of parthenocarpy. In addition, some small degree of parthenocarpy was still found when stigmas started to develop anthocyanin (DAP = 3). This indicates that some flowers had not yet been pollinated.

We target candidate genes from only the auxin signaling pathway. After confirming by RT-PCR and qRT-PCR expression, *EgGH3.8*, *EgGH3.1*, *EgARG7*, *EgTAA3* and *EgFMO1* were clearly differentially expressed between the treatments and controls. *EgGH3.8* and *EgGH3.1* were found to be expressed more in the treatments while *EgARG7*, *EgTAA3* and *EgFMO1* were found to be expressed more in the controls. These candidate genes were reported to be involved in parthenocarpy and fruit development in the following studies. Most early auxin response genes were classified into three families, including *AUX/IAAs*, *GRETCHEN HAGEN3s* (*GH3*) and *SMALL AUXIN UP RNAs* (*SAURs*) (Hagen & Guilfoyle, 2002). Several RNA seq studies revealed that *AUX/IAAs*, *GH3* and *SAURs* are involved in parthenocarpy. Li et al. (2014) performed RNA seq and ovary transient expression and proposed 14 putative genes in auxin, cytokinin and GA regulations that were involved in the development of parthenocarpic cucumber (*Cucumis sativus*) fruits. The predicted parthenocarpic genes in auxin regulation included *AUX/IAAs*, *GH3* and *SAURs*. Also, several DEGs associated with auxin, such as *AUX/IAAs*, *GH3* and *SAURs* were identified from induced parthenocarpic fruits of *Siraitia grosvenorii* (Tu et al., 2017). The *GH3* family of enzymes are involved in a range of hormone-dependent processes, including fruit development, root growth, flowering, hypocotyl growth and disease resistance (Chen et al., 2010). *GH3* family genes encode for an IAA-amido synthetase that regulates free IAA level. They function to maintain auxin homeostasis by reversibly conjugating excess IAA with amino acids to prevent IAA accumulation (Chen et al., 2010; Westfall et al., 2016). The *GH3* family genes are found to be both down and up-regulated during anthesis. In this study, *EgGH3.1* and *EgGH3.8* were both up-regulated in the auxin treated pistils, which developed into artificially parthenocarpic oil palm fruits. We suggest that the up-regulation of these genes may be a result of the excess auxin during the treatment at anthesis. Kang et al. (2013) has found that *GH3* family genes, such as *GH3.1* and *GH3.17*, were both up and down-regulated during early fruit development of strawberry, depending on the type of fruit tissues. Interestingly, probable IAA-amido synthetase *GH3.1* was down-regulated in flower buds of naturally parthenocarpic eggplants that were collected 1 day before anthesis (Chen et al., 2017). This result may be because *GH3.1* expression is not necessary when there are no excess IAA, which would be the case in the flower buds before pollination. The natural parthenocarpy in this eggplant may be induced from other factors during anthesis, which may or may not be auxin-related.

In this study, several genes in the auxin signaling pathway, including *EgGH3.8*, *EgGH3.1*, *EgARG7*, *EgTAA3* and *EgFMO1*, have been revealed as potential genes that would be instrumental for producing parthenocarpic oil palm with higher oil content. Moreover, these genes could also be targeted in other flowering plant species to increase fruit quality, fruit shelf-life and other characteristics that are related to the absence of seeds. Although the influence of auxin on parthenocarpic development has been reported since the early twentieth century (Gustafson, 1937, 1939), the molecular mechanisms are still studied extensively today. The study of parthenocarpic mechanism can also increase our understanding of the fruit setting process, which occurs at the early stage of fruit development. Gaps still remain in proposed auxin response model (Ariizumi, Shinozaki & Ezura, 2013; De Jong, Mariani & Vriezen, 2009; Tang et al., 2015). The potential genes above could help to fill in some of these gaps, especially concerning fruit set initiation and development, both of which are tightly regulated in a complex network. Furthermore, these genes could be targeted for the development of transgenic parthenocarpic fruits, like in the previous work done in eggplant and tomato (Acciarri et al., 2002; Rotino et al., 2005, 1998). Consequently, this work can add to basic knowledge of auxin regulating genes involved in the fruit development process and perhaps these genes may be exploited for parthenocarpic fruit production in the near future.

## Conclusions

We propose five candidate genes that are potentially involved in parthenocarpy, based on their differential expression between auxin-treated and untreated oil palm samples. Those candidate genes included probable IAA-amido synthetase GH3.8 (*EgGH3.8*), IAA-amido synthetase GH3.1 (*EgGH3.1*), IAA induced ARG7 like (*EgARG7*), tryptophan amino transferase-related protein3-like (*EgTAA3*) and flavin-containing monooxygenase 1 (*EgFMO1*). There was a limitation in the amount of female inflorescences produced by each oil palm plant. This affected our experimental design for both treatments and controls by limiting the amount inflorescences used as replicates. Each oil palm inflorescence for each oil palm plant were produced in different times resulting a limitation in the amount inflorescences at the same pollination stage in the same plant. Consequently, we designed to compare RNA seq from the same stage but different oil palm plants as the treatments or controls at the given pollination stages. This can affect the amount and the reliability of DEGs. For example, RNA seq revealed probable IAA-amido synthetase GH3.8 (*EgGH3.8*) from three out of five comparisons, flavin-containing monooxygenase 1 (*EgFMO1*) and IAA-amido synthetase GH3.1 (*EgGH3.1*) from two comparisons while IAA induced ARG7 like (*EgARG7*) and tryptophan amino transferase-related protein3-like (*EgTAA3*) were revealed from only one comparison. However, if we can overcome these limitations, more candidate DEGs involved in parthenocarpy may be revealed.

## Supplemental Information

10.7717/peerj.5975/supp-1 Supplemental Information 1 Examples of four oil palm female inflorescences, with different anthesis/pollination stages, which were selected for the first day of auxin treatment. Day after pollination (DAP) = 0 means that the stigma has not yet opened. DAP = 1 means that the stigma has been opened for one day. DAP = 2 means that the stigma has been opened for two days. DAP = 3 means that the stigma has been opened for three days.

10.7717/peerj.5975/supp-2 Supplemental Information 2 A comparison of stigmas from auxin-treated pistils and fruits with control pistils and fruits. Example of stigmas from auxin-treated Inflo.3T, first sprayed at DAP = 1, which were red and fresh for two weeks, compared with normal stigmas from Inflo.4C, which were black and dry since DAP = 5 (A). The stigmas of Inflo.1T fruits, which were first sprayed at DAP = 0 were still red and fresh for 6 weeks after anthesis, compared with Inflo.1C fruits without auxin treatment (B).

10.7717/peerj.5975/supp-3 Supplemental Information 3 KEGG pathway classification for the 83 nonredundant DEGs. The number of up-regulated DEGs are shown in the positive y-axis while the number of down-regulated DEGs are shown in the negative y-axis.

10.7717/peerj.5975/supp-4 Supplemental Information 4 Details of oil palm inflorescences. DAP = Day After Pollination.

10.7717/peerj.5975/supp-5 Supplemental Information 5 Details of oil palm inflorescences that were used for the auxin treatment, which was first spraying on DAP = 0 and DAP = 1 compared with the controls without auxin treatment. The female flowers (pistils) of different stages of each oil palm number were collected for RNA extraction. The pistil sample collection was performed for 3 consecutive days, Day 1, 2 and 3. Samples without auxin treatment are marked as x, with auxin treatment for 24 hours are marked as / and with auxin treatment for 48 hours are marked as //. The samples without auxin treatment included Inflo.1T/NA (/NA = No auxin treatment), Inflo.1C/NA, Inflo.2C/NA1, Inflo.2C/NA2 and Inflo.4C/NA. The samples with auxin treatment included Inflo.1T/WA (/WA = With auxin treatment), Inflo.6T/WA and Inflo.4T/WA. NS represent no sample because mRNA isolation from these samples was not successful.

10.7717/peerj.5975/supp-6 Supplemental Information 6 Eight comparisons of differentially expressed genes (DEGs) between the treatments and controls revealed both up-regulated and down-regulated DEGs. The DEGs were also categorized by KEGG.

10.7717/peerj.5975/supp-7 Supplemental Information 7 Details of primers that were used to determine the expression of six candidate genes by RT-PCR and qRT-PCR.

10.7717/peerj.5975/supp-8 Supplemental Information 8 Eight comparisons of differentially expressed genes (DEGs) between the treatments (Tt) and controls (Ct) revealed both up-regulated DEGs (positive number of log2FoldChange) and down-regulated DEGs (negative number of log2FoldChange). The 1^st^ comparison was between Inflo.1T/WA (Tt) and Inflo.1C/NA (Ct). The 2^nd^ comparison was between Inflo.1T/WA (Tt) and Inflo.1T/NA (Ct). The 3^rd^ comparison was between Inflo.6T/WA (Tt) and Inflo.2C/NA2 (Ct). The 4^th^ comparison was between Inflo.6T/WA (Tt) and Inflo.2C/NA1 (Ct). The 5^th^ comparison was between Inflo.6T/WA (Ct) and Inflo.4C/NA (Ct). The 6^th^ comparison was between Inflo.4T/WA (Tt) and Inflo.2C/NA2 (Ct). The 7^th^ comparison was between Inflo.4T/WA (Tt) and Inflo.2C/NA1 (Ct). The 8^th^ comparison was between Inflo.4T/WA (Tt) and Inflo.4C/NA (Ct).

10.7717/peerj.5975/supp-9 Supplemental Information 9 Supplemental data of six candidate gene sequences in auxin signaling pathway that were used to design primers for RT-PCR and qRT-PCR confirmation. The candidate genes included *EgTAA3*, *EgGH3.8*, *EgGH3.1*, *EgARG7*, *EgSAUR71* and *EgFMO1*. These genes were differentially expressed between auxin-treated and untreated pistils of oil palm. These sequences are available on the NCBI database (https://www.ncbi.nlm.nih.gov/).

10.7717/peerj.5975/supp-10 Supplemental Information 10 Original electrophoretic images for Fig.3 RT-PCR confirmation.

## Additional Information and Declarations

### Data Availability

The following information was supplied regarding data availability: The raw data, candidate gene sequences and original electrophoretic images are available as Supplemental Files. DNA sequences can be found at NCBI under accession numbers: XM_019853925.1, XM_019853925.1, XM_010943300.2, XM_010938255.2, XM_010940836.2, XM_010942329.2, XM_010916766.2.
