# Supplementary figures and images for: Transcriptome analysis of oil palm inflorescences revealed candidate genes for an auxin signaling pathway involved in parthenocarpy
Source: PeerJ. 2018 Dec 17;6:e5975. doi: 10.7717/peerj.5975 (PMC6301279; doi:10.7717/peerj.5975)

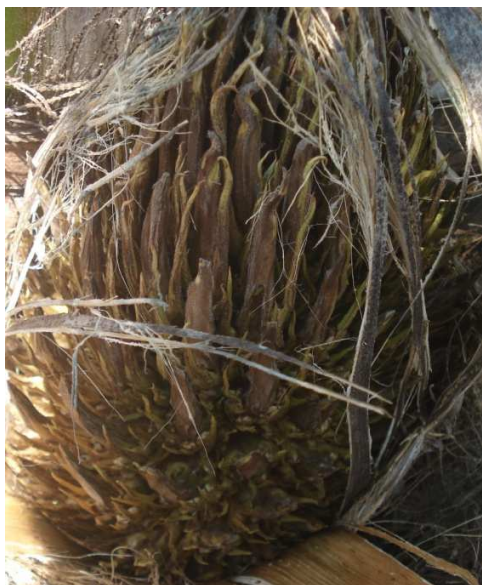

Inflo.4T at DAP = 0

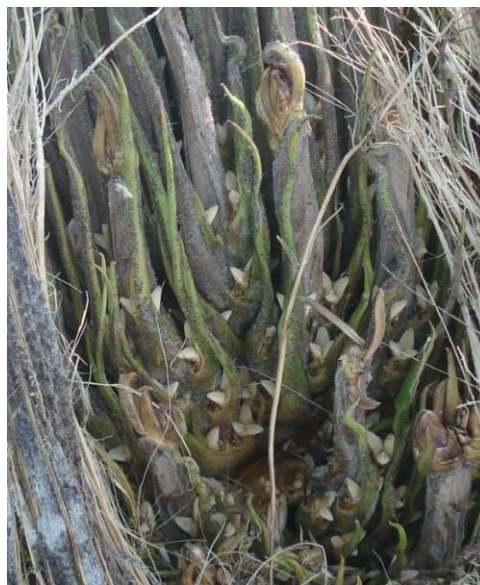

Inflo.8T at DAP = 1

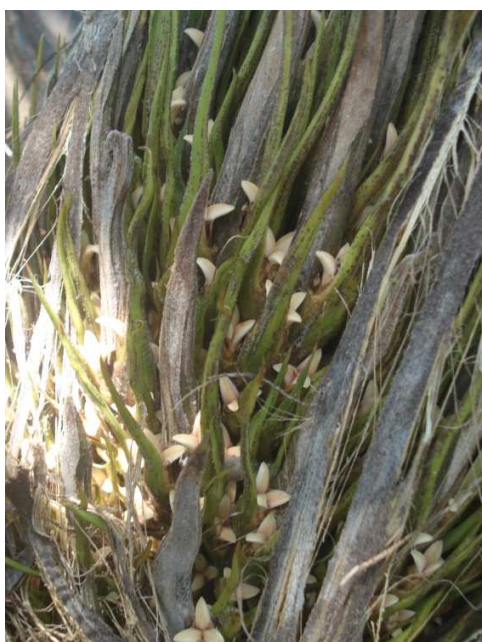

Inflo.11T at DAP = 2

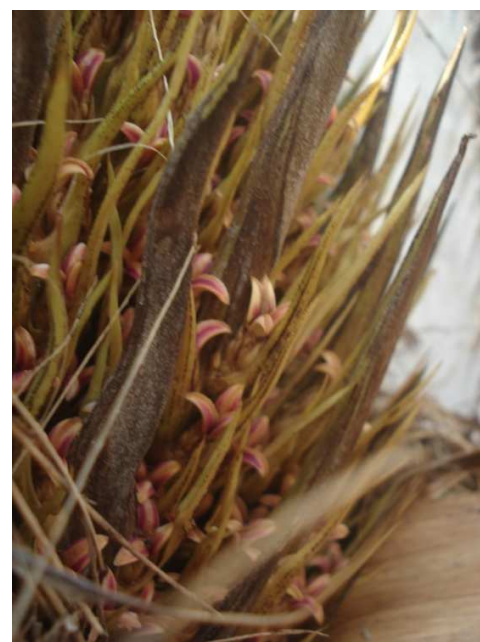

Inflo.13T at DAP = 3

Supplement: Supplemental Information 1 — Day after pollination (DAP) = 0 means that the stigma has not yet opened. DAP = 1 means that the stigma has been opened for one day. DAP = 2 means that the stigma has been opened for two days. DAP = 3 means that the stigma has been opened for three days. [file peerj-06-5975-s001.pdf]

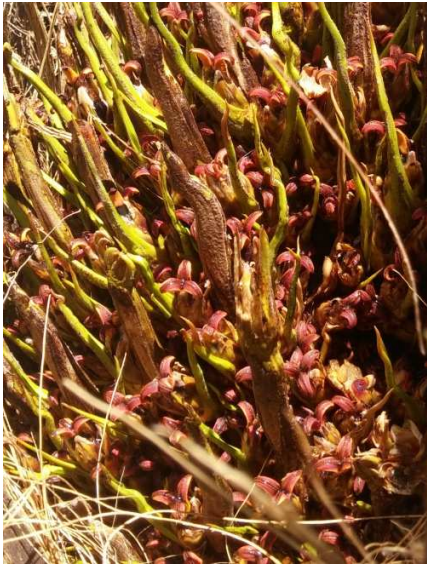

Inflo.3T

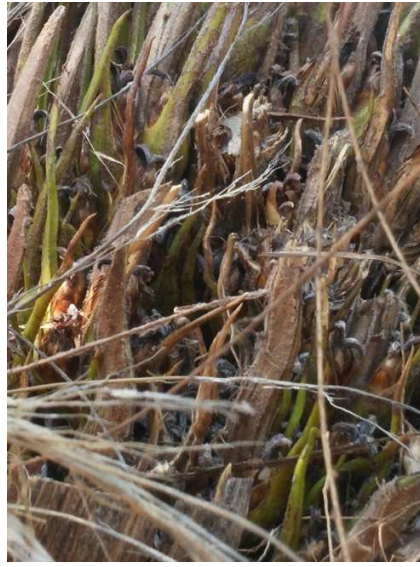

Inflo.4C

A

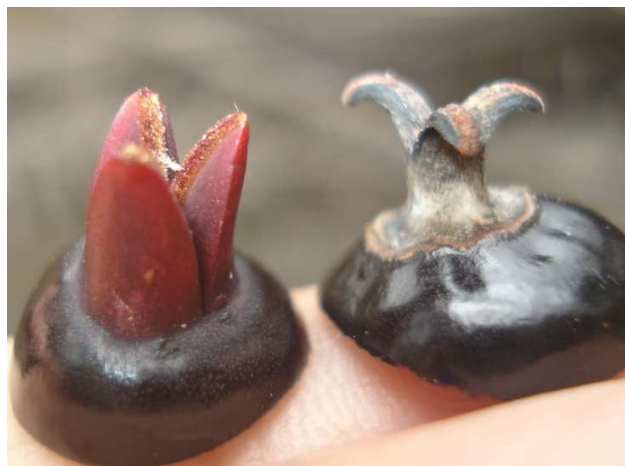

Inflo.1T fruit

Inflo.1C fruit

B

Supplement: Supplemental Information 2 — Example of stigmas from auxin-treated Inflo.3T, first sprayed at DAP = 1, which were red and fresh for two weeks, compared with normal stigmas from Inflo.4C, which were black and dry since DAP = 5 (A). The stigmas of Inflo.1T fruits, which were first sprayed at DAP = 0 were still red and fresh for 6 weeks after anthesis, compared with Inflo.1C fruits without auxin treatment (B). [file peerj-06-5975-s002.pdf]

KEGG pathways for the nonredundant DEGs

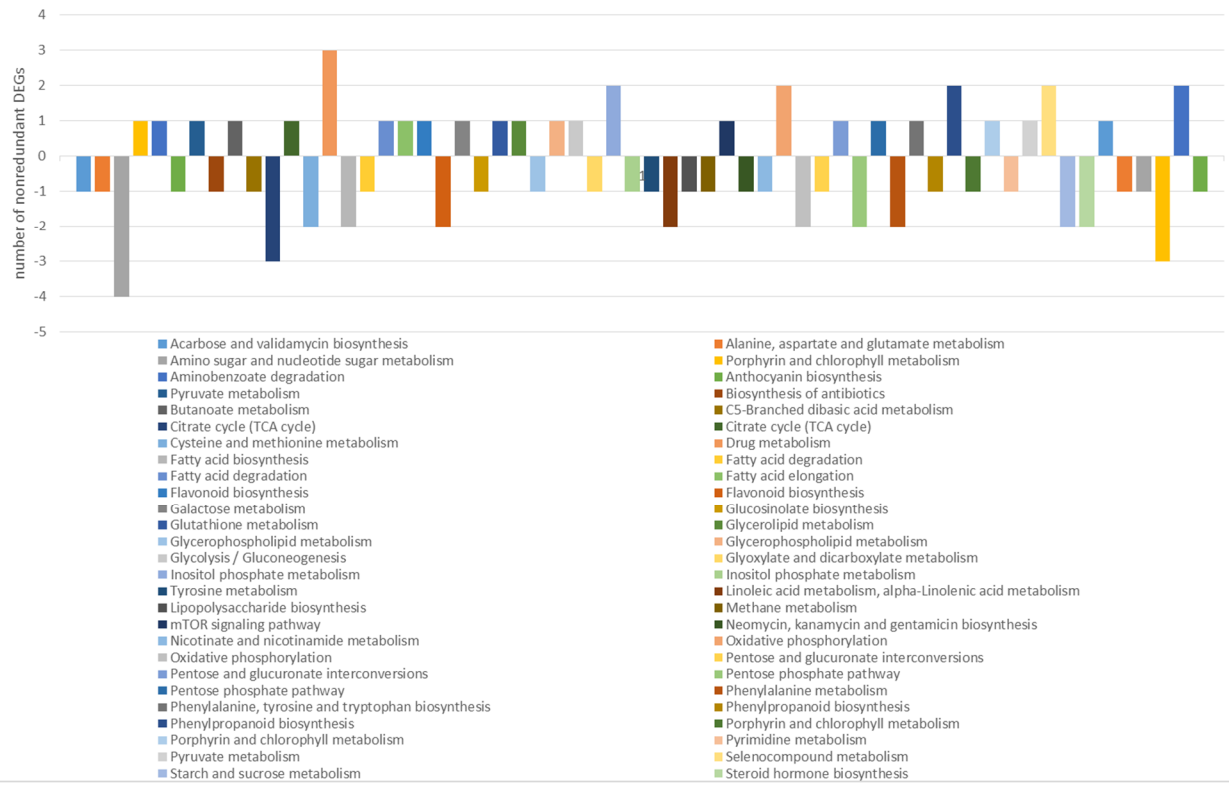

Supplement: Supplemental Information 3 — The number of up-regulated DEGs are shown in the positive y-axis while the number of down-regulated DEGs are shown in the negative y-axis. [file peerj-06-5975-s003.pdf]
