# Supplementary material for: Transcriptome analysis of oil palm inflorescences revealed candidate genes for an auxin signaling pathway involved in parthenocarpy
Source: PeerJ. 2018 Dec 17;6:e5975. doi: 10.7717/peerj.5975 (PMC6301279; doi:10.7717/peerj.5975)
Supplement: Supplemental Information 4 [file peerj-06-5975-s004.docx]

Table S1

| Treatment / control group | Inflorescence name | Oil palm tree |
| --- | --- | --- |
| Treatment on DAP = 0 | Inflo.1T | C2-21 |
|  | Inflo.2T | C2-09 |
| Treatment on DAP = 1 | Inflo.3T | C1-08 |
|  | Inflo.4T | C1-18 |
|  | Inflo.5T | C2-15 |
|  | Inflo.6T | C2-19 |
| Treatment on DAP = 2 | Inflo.7T | C1-05 |
|  | Inflo.8T | C1-16 |
|  | Inflo.9T | C2-14 |
|  | Inflo.10T | C2-22 |
| Treatment on DAP = 3 | Inflo.11T | C1-03 |
|  | Inflo.12T | C1-09 |
|  | Inflo.13T | C1-22 |
| Control for DAP = 0 | Inflo.1C | C2-21 |
| Control for DAP = 1, 2 and 3 | Inflo.2C | C1-16 |
|  | Inflo.3C | C2-11 |
|  | Inflo.4C | C2-22 |
