# Supplementary material for: Transcriptome analysis of oil palm inflorescences revealed candidate genes for an auxin signaling pathway involved in parthenocarpy
Source: PeerJ. 2018 Dec 17;6:e5975. doi: 10.7717/peerj.5975 (PMC6301279; doi:10.7717/peerj.5975)
Supplement: Supplemental Information 5 — The female flowers (pistils) of different stages of each oil palm number were collected for RNA extraction. The pistil sample collection was performed for 3 consecutive days, Day 1, 2 and 3. Samples without auxin treatment are marked as x, with auxin treatment for 24 hours are marked as / and with auxin treatment for 48 hours are marked as //. The samples without auxin treatment included Inflo.1T/NA (/NA = No auxin treatment), Inflo.1C/NA, Inflo.2C/NA1, Inflo.2C/NA2 and Inflo.4C/NA. The samples with auxin treatment included Inflo.1T/WA (/WA = With auxin treatment), Inflo.6T/WA and Inflo.4T/WA. NS represent no sample because mRNA isolation from these samples was not successful. [file peerj-06-5975-s005.docx]

**Table S2**

| Treatment group | Inflorescence name | Oil palm tree | Name of pistil samples collected at | | | Parthenocarpy degree |
| --- | --- | --- | --- | --- | --- | --- |
|  |  |  | Day 1 | Day 2 | Day 3 |  |
| Auxin treated on DAP = 0 | Inflo.1T | C2-21 | x | / | // | 100% seedless fruits |
|  |  |  | Inflo.1T/NA | Inflo.1T/WA | |  |
|  | Inflo.1C | C2-21 | x | x | x | 100% seeded fruits |
|  |  |  | NS | Inflo.1C/NA | |  |
| Auxin treated on DAP = 1 | Inflo.6T | C2-19 | x | / | // | 50% seedless fruits |
|  |  |  | NS | Inflo.6T/WA | |  |
|  | Inflo.4T | C1-18 | x | / | // | 90% seedless fruits |
|  |  |  | NS | Inflo.4T/WA | |  |
|  | Inflo.2C | C1-16 | x | x | x | 100% seeded fruits |
|  |  |  | Inflo.2C/NA1 | | Inflo.2C/NA2 |  |
|  | Inflo.4C | C2-22 | x | x | x | 100% seeded fruits |
|  |  |  | NS | Inflo.4C/NA | |  |
