# Supplementary material for: Transcriptome analysis of oil palm inflorescences revealed candidate genes for an auxin signaling pathway involved in parthenocarpy
Source: PeerJ. 2018 Dec 17;6:e5975. doi: 10.7717/peerj.5975 (PMC6301279; doi:10.7717/peerj.5975)
Supplement: Supplemental Information 6 [file peerj-06-5975-s006.docx]

Table S3

| Comparison Order | Treatments | Controls | Up-regulated DEGs | Down-regulated DEGs | Total DEGs | KEGG-categorized genes |
| --- | --- | --- | --- | --- | --- | --- |
| 1 | Inflo.1T/WA | Inflo.1C/NA | 30 | 109 | 139 | 15 |
| 2 | Inflo.1T/WA | Inflo.1T/NA | 72 | 87 | 159 | 16 |
| 3 | Inflo.6T/WA | Inflo.2C/NA2 | 167 | 118 | 285 | 18 |
| 4 | Inflo.6T/WA | Inflo.2C/NA1 | 140 | 95 | 235 | 16 |
| 5 | Inflo.6T/WA | Inflo.4C/NA | 141 | 105 | 246 | 26 |
| 6 | Inflo.4T/WA | Inflo.2C/NA2 | 80 | 40 | 120 | 14 |
| 7 | Inflo.4T/WA | Inflo.2C/NA1 | 93 | 49 | 142 | 5 |
| 8 | Inflo.4T/WA | Inflo.4C/NA | 117 | 98 | 215 | 26 |
| Total |  |  | 840 | 701 | 1541 | 136 |
