# Supplementary material for: Transcriptome analysis of oil palm inflorescences revealed candidate genes for an auxin signaling pathway involved in parthenocarpy
Source: PeerJ. 2018 Dec 17;6:e5975. doi: 10.7717/peerj.5975 (PMC6301279; doi:10.7717/peerj.5975)
Supplement: Supplemental Information 7 [file peerj-06-5975-s007.docx]

Table S4

| Gene abbreviation | Genes | Gene cds | NCBI accession number | Forward primer (5′-3′ direction) | Reverse primer (5′-3′ direction) | Expected product size (bp) |
| --- | --- | --- | --- | --- | --- | --- |
| *EgFMO1* | flavin-containing monooxygenase 1 (cds 26665) | cds26665 | XM_019853925.1 | TTGCCACCCGGTAGTAAGAG | CAAGAAGCCTTTCTCCAACG | 174 |
| *EgSAUR71* | auxin-responsive protein SAUR71-like | cds32638 | XM_010943300.2 | GATTGGCGATGAAGAGGTTG | GGTCGTAGCCGTACTCCTGA | 249 |
| *EgSAUR71* | auxin-responsive protein SAUR71-like | cds32638 | XM_010943300.2 | GCTCAGGAGTACGGCTACGA | GACACGGGTCGTTAGAAAGG | 220 |
| *EgGH3.8* | indole-3-acetic acid-amido synthetase GH3.8 | cds27914 | XM_010938255.2 | GTCCAAAAGAAGGTGCTTGC | GGAGCTGGTGAGGAACTCAG | 219 |
| *EgGH3.1* | indole-3-acetic acid-amido synthetase GH3.1 | cds30335 | XM_010940836.2 | CACTCCCGTCATGAACCTCT | GTAGGGCTGGTGATGACGTT | 186 |
| *EgARG7* | indole-3-acetic acid induced ARG7 like | cds31450 | XM_010942329.2 | GTTCTCAAGCATGCAGTTCG | GCAGCTCAAAGACACCACAA | 210 |
| *EgTAA3* | trytophan amino transferase-related protein3-like | cds7351 | XM_010916766.2 | AAGCAGTGCAGTTGTTGTGG | GGAGAAAGAGCATGGACTGC | 198 |
