# Supplementary material for: Transcriptome analysis of oil palm inflorescences revealed candidate genes for an auxin signaling pathway involved in parthenocarpy
Source: PeerJ. 2018 Dec 17;6:e5975. doi: 10.7717/peerj.5975 (PMC6301279; doi:10.7717/peerj.5975)
Supplement: Supplemental Information 9 — The candidate genes included EgTAA3, EgGH3.8, EgGH3.1, EgARG7, EgSAUR71 and EgFMO1. These genes were differentially expressed between auxin-treated and untreated pistils of oil palm. These sequences are available on the NCBI database (https://www.ncbi.nlm.nih.gov/). [file peerj-06-5975-s009.docx]

Supplemental data of six candidate gene sequences in auxin signaling pathway that were used to design primers for RT-PCR and qRT-PCR confirmation. The candidate genes included *EgTAA3*, *EgGH3.8*, *EgGH3.1*, *EgARG7*, *EgSAUR71* and *EgFMO1*. These genes were differentially expressed between auxin-treated and untreated pistils of oil palm. These sequences are available on the NCBI database (https://www.ncbi.nlm.nih.gov/).

**Sequence 1 was used for designing RT-PCR primers for *EgTAA3***

XM_010916766.2 PREDICTED: Elaeis guineensis tryptophan aminotransferase-related protein 3-like (LOC105040296), mRNA TGTTTTTCTTTTTTCAAAAAAAAAATATTTTCGAAAAGATTACTTTTCAAAGCTTGCATTTTCTGGTGAAAGATGGAATAACTTGGAAGCAATGGCTTCAACTGCAGTCTACTTTGAAATATATACGTGCTAATGGTTAGGTCATTCTTGACGATGTGAACACAGTTCAGAAACGTAAGCCATAAAGATTGGTCAAAGGAACCATACCAAGGAAATGAGATTATTATCATATCATCCGTGCGTCTTGAAATAAGGGACATGACAGCTGACTTTTTCTATGGTTTATTGTTCATCCCTACAAACTTAAATCTTTCCTCCCCCTTGGTCTGAGGATCATATTGACTGCGCGCTTCAAAGTGGTGATCCTAATCTAGTGTTCGAATTGAACACCATCTGTTTATTGACCGCGCGCTTCCAAGTGGTGATCCTAATCCAGTGTTCAAATTGAACACCTGCTCAAACTTTTCGCCTGTTGAATGGTTTCTCTGCAGATCGGTGATTGTTCGAAGCAAGCTAAAGCTGCGAGTATAAAAAGGACCTCATGGAACTGCAAACTAGCGGAAATAAAACAAAGAAATATGGATTCGTCCCAACTCTCTATCAAACTGATAGAAGGGCCCAAAGCGCCCAATACCAAGGCCTATAACTTCAGTTACCTCTTCTGTCTCTCCTTGTTAATATGTTCTCCCTGTTTGAATTTCTTCTTCATCTCTAGAGCTCTTTTCCGATCACCTGAGCCAAGCTGGAGCAGCAGAGCAGCCACGGAAGCTGAAGCTGTGGCAGCTCGCTCGTGTTCTGGGCATGGATCAGCCTTTGTGGACGGGATTCTTGTCGACGGGAAGCATGTCTGTGAGTGCAACGCATGTTATAGAGGCCCGGATTGCTCGGAATTTGTGCAGGGTTGTGCTGCTGATGCTGAAAGTGGCGATCCCCTGTTTCTGGAGCCATACTGGAGGCAGCATGCAGCAAGCAGTGCAGTTGTTGTGGCCGGTTGGCACCGTATGAGCTACCAAACAGATGGTGATTACTTCATCTCTATCGAACTCGAAAGACACATTCGAAAACTGCATTCACATGTAGGAAATGCAATCACCGATGGGAGATTCATCATCTTTGGGGCTGGATCCACGCAGCTGATCAACGCAGCAGTCCATGCTCTTTCTCCAAACAGCACAGTATCATCCCCTGCTAGTGTTGTTGCATCCGTCCCCTACTACATGGTTTATAGAACGCAGACCGAGTTCTTCAACTCCCGAGCGTATGAATGGAAAGGGGTAACATCAAAATGGGTCGACAAGTTGAACTCATCTGCGCCAAATTTTATCGAATTTGTGACATCTCCCAACAACCCAGATGGGCGCATGCAGCAACCGATTCTTCCAGGTTCATCCGTGATTTACGACCACGCCTACTACTGGCCTCACTTCTCCGCAATCAAAGCTCCAGCTGATGGAGATCTCATGCTCTTCACCATCTCCAAGCTCACAGGTCACGCCGGTAGCAGATTTGGGTGGGCGATTGTGAAGGATGAGGGAGTGTACAAGAGAATGCTGACCTACCTGGGAATCAACACGATGGGTGTTTCCAGGGATACTCAGCTAAGAAGTCTGAAGCTCCTGAAAGCAGTGCTGGGTGAAGAGAGAGGGGCGGCAGGGAGCATCTTTGAGTTTGCATTTAGGACCATGAGGGAAAGGTGGCACAAATTGAACAAGCTTGTGTCGTCCTCCGACCGGTTCTCCCTCCAACAAACGCGTCCTGAGTACTGCAACTATTTTGGAAAGATCAGGGACCCTTCTCCAGCTTACGCTTGGTTGAAGTGCGAGAGGGAGGAAGACAAAAACTGCTATGCTGTATTGAGAGCTGGAGGAATAATCTCCCGCTCGGGGTACAAATTTGGAGCAGATGGCCGCTACACTCGTCTGAGCCTCATAAGGTCGCAAGACGACTTCGATTTGTTGATTCGACGGATGGATGCTTTGATCACCAAGGAGAAAGCCAGAAATATGTGAGAAGGATTGAGATAGAGATCGTACGGGGAAAACATTTATAGAAGAACACCTTCTTTTGGATATTCAATATTTATCACCTCATACATT

**Sequence 2 was used for designing RT-PCR primers for *EgGH3.8***

XM_010938255.2 PREDICTED: Elaeis guineensis probable indole-3-acetic acid-amido synthetase GH3.8 (LOC105056155), mRNA GCCACCTCCTTTCCCCGGCGTTCCATCTCTCGAGCACAGAGACTAATTGACAGGGGCCAAATGGCCGTGGAAACCACCACTTACACATCCCTCGGCCCGGCCGCCGACGAGAGGGATGAAGAAAAGCTCCGATTCATCGATGAGATGACCGCCAATGCCGACGCGGTCCAAAAGAAGGTGCTTGCCGAGATCCTGAGCCGGAACGCTGAGACTGAGTACCTCCAGAGGTATAACCTCGGCGGCGCCACTGACCGTGCCACGTTTAAGGCTAAGATCCCCATGGTCACCTATGAGGACCTTCAGCCGGAGATCCAGCGTATTGCTAACGGTGACCGCTCTCCCATCCTCTCTGCCTACCCTATCTCTGAGTTCCTCACCAGCTCCGGGACGTCGGCCGGTGAACGGAAGCTGATGCCGACGATCAAAGAAGAGCTGGACCGCCGGCAGCTCCTCTACAGCCTTCTAACGCCCGTCATGAATCTCTATGTGCGGGGGCTGGACAAGGGAAAGGCCCTCTACTTTTACTTCGTGAAGTCGGAGACCAGGACCCCCGGCGGGCTCTTGGCGCGGCCGGTGCTGACGAGCTACTACAAGAGCGAGCACTTCAAGAGCCGGCCCTACGACCCCTACAACGTCATCACGAGCCCCACCCCCGCCATCCTCTGCGCCGACTCCTTCCAGAGCATGTACGTCCAGATGCTCTGCGGCCTCCTCCACCGCCTCCAAGTCCTCCGTGTCGGCGCCGTCTTCGCCTCCGGCCTCCTCCGCGCCATCCGCTTCCTGCAGCTCCACTGGGGGGAGCTCGCCCGCGACATCGCCGCCGGCTCGCTCAGCCCCAAGATCACCGACCCCTCGGTCCGGGACTCGGTCGCCGACGTCCTGAAGCCCAACACCGAGCTCGCCGAGTTCATTAGAGCCGAGTGCTCGACCGGGGAGTGGGCGGGGATCGTTACCAGGATGTGGCCCAACACCAAATATCTGGACGTGATCGTGACGGGGGCCATGGCACAGTACATCCCCACCTTGGAGTACTACAGCGGGGGGCTCCCCATGGCGTGCACCATGTACGCCTCCTCCGAGTGCTACTTCGGCCTCAACCTGCGGCCTATGTGCAAGCCCTCCGAGGTCTCCTACACCATCATGCCCAACATGGCCTACTTCGAGTTCCTCCCTCTCGACCTTGCCGCCGGCGGCGCGTGCCAGGGGGAGGAGCTGGTGGACCTGGCACGCGTGGAAGTGGGCAAGGAGTACGAGCTGGTGATCACCACCTACGCGGGGCTCTACCGGTACCGGGTCGGCGACATCCTCCGGGTGACGAGCTTCCACAACGCGGCCCCACAGTTCCGGTTCGTGCGGCGGAAGAACGTGCTGCTGAGCATCGAGTCGGACAAGACCGACGAGGCGGAGCTCCAGAAGGCGGTGGAGAGAGCGTCGGAGCTCCTCGAGCCGTGGAACGCCACCGTGGTGGAGTACACCAGCCACGCCTCGACGAAGAGCATCCCGGGGCACTACGTCGTCTACTGGGAGCTCCTGGTGAAGGAGTCCACGGGTGAAGGGAGAATGCCGTCCGGGGAGGTGATGGGGCGTTGCTGTCTGGCGATGGAGGAGGCGCTGAACTCGGTGTACCGGCAGAGCCGGGTGGCGGACGGGTCGATCGGGGCGCTGGAGATCCGGGTGGTGAGGGGAGGGACCTTCGAAGAGCTCATGGACTACGCCATCTCGAGGGGGGCGTCCATCAACCAGTACAAGGTCCCGCGCTGCGTCAACTTCCCCCCGATCTTGGACCTGCTCGACTCCCGGGTCGTCTCCACCCACTTCAGCCCCACCCTCCCCACCTGGAGCCCCCACCGCGCCACCTAGAACGTGGGCCCCACGTGTGTGTTTCCGTGGCGCTGACCGACCGACCAACTTACCTTCCCTGTCTTTTCCTAGTCAACGGCCGTGCCGGGGACGTAGCTGCAATTTGTTTCCATCCCGTGGAAGACGGCCTATTACTTTCTTTTCTCCTCTATTCCTTTGTTTAAAATTAAAAAAAAAAGAAAAAAAAGAAGAAGGAGAACATCATCTACTTTATTAATGTCAAATAGTGGCGCTTTTTT

**Sequence 3 was used for designing RT-PCR primers for *EgGH3.1***

XM_010940836.2 PREDICTED: Elaeis guineensis probable indole-3-acetic acid-amido synthetase GH3.1 (LOC105058058), mRNA TGCTCGTACCAAGCAAAGATTAATAAACCAAATGGCCGTGGAAACCCCCATCCCCACCACGTCCTCCACATCCCTCGGCCCGGCCACGAACGAGAAAGATGCCGAGAAGCTCCGTTTTATCGATGAGATGACCGCCAACGCGGACACGGTCCAGGAGAAGGTGCTGGCCGAGATCCTTAGCCGGAACGCCGAGACCGAGTACGTCCAGAGGTACAAGCTTGGCAGTGCCACCGACCGTGCCACCTTTAAGGCTAAGATCCCCATGGTCACCTATAAGGACCTCCAGCCGGAGATCCAGCGCATCGCCAACGGAGACTGCTCTCCCATCCTCTCCGCCCTCCCCATCTCCGAATTCCTCACGAGCTCCGGGACATCGGCCGGTGAACGGAAGCTGATGCCTACCATCAAAGAAGAGCTGGACCGCCGGCAGCTCCTCTACAGCCTTCTCACTCCCGTCATGAACCTCTACGTGCCGGGACTGGACAAGGGGAAGGCCCTCTACTTTTACTTCGTGAAGTCGGAGACCAAGACTCCCGGCGGACTGTTGGCGCGGCCGGTGCTGACGAGCTACTACAAGAGCGAGCACTTCAAGAGCCGGCCATACGACCCCTACAACGTCATCACCAGCCCTACCCCCGCCATCCTCTGCGCTGACTCCTTCCAGAGCATGTACGTCCAAATGCTCTGCGGCCTCCTCCACCGCCTCGAAGTCCTCCGCGTCGGCGCCATTGAGGATCACGAGAAGTCATCACACTTCATGAATTACACAACGATCCTCACAAAGCTGAGAGAACTCTCTTGA

**Sequence 4 was used for designing RT-PCR primers for *EgARG7***

XM_010942329.2 PREDICTED: Elaeis guineensis indole-3-acetic acid-induced protein ARG7-like (LOC105059115), mRNA ATGGCCAAGGCAAACTTCATCAGGAGAAGCAGTGTTCTCAAGCATGCAGTTCGAGTGCTGCAGAGGATCAGTCTTCCATTATCAAAGATGAAGCCTGCTCCTCCAATGCGGGGTAATTTATCCGGTGAGTTCGAGGAGGCTGACAGAACTGGAATGGTGCCGGAGGATGTGAAGGAAGGCCACTTTGCAGTGGTGGCAGTCTTTGATGAGAAGCCAAAAAGATTTGTGGTGTCTTTGAGCTGCCTCTCACATCCTATGTTTCTGAGGTTATTGGAGTTGGCAGAAGAGGAGTTTGGCTTCCGGCAAGAGGGGCCCCTTGCTGTACCCTGCCGGCCGAGTGACCTGGAGAAGATAATCCTAGAGCTATGAGGAAGAGGACAAGTTTCATGTCAATTGGTTCTCCTCCATCACTACTTTTCTCTTTATATATATACATATTTATGTAGTTCTGTTGATCTAATGACAAGATCAAGGATCACATTGGATCCTATCAACCAGAAATCAGAGAAGCATGTACTATGTGGTATGCTACTGTTAGGCCAGCAATTAAAATGCGAATGATGATTGTAGCATATATATATATGGATGATTAAGATCA

**Sequence 5 was used for designing RT-PCR primers for *EgSAUR71***

XM_010943300.2 PREDICTED: Elaeis guineensis auxin-responsive protein SAUR71-like (LOC105059840), mRNA

CCTCAGCCTACCTCCCCCCAACTCCTCCCCACGCTCTCCACGCCTATCTCTCTCTCTCTCTCTCTCGATTTGTCTCGAGATTGGCGATGAAGAGGTTGATCAGACGGCTGTCGAGGGTGGCCGACTCGTCGCAGTGCACGCCACCGCGGGAGGCGAAAGAGGAAGGAGGCGGCGGCGGCGGAGGGCGGCGCCGGAAGGTGCCGGAGGGGTACGTGCCGGTGTACGTGGGGGAGGAGATGAAGCGGTTCGAGGTCCGGGCGGAGCTACTGGGCCGGCCGGCCTTCGTCGAGCTCCTCCGCCGGTCCGCTCAGGAGTACGGCTACGACCAGCGCGGCGTCCTCCGCATCCCCTGCCCCGTCCCTCTCTTCCGCCAGGTCCTCTACTTTCTTGCCTCCGGCGCCGACGCGGGCGGCGATCTCGCCGTCGAGGAGCTCTTCCGCTCGCTCCCCGAGGAGATCCCGTCCGGCTCCCCTTCGTCGGGAGAAGCGCCTGTTTCCCATTTTGCCCTTTCTAACGACCCGTGTCCGTATAACGCAGTCTGAGGTGAATGAGGTGGAGGGCAATCTGGAGATTTTATTTTACTCTTCCTTTTTCTTTTTGCAATGGTAGTATGTAGGCAGGGGGAATCTAGCGGGATGGAGGCAGGGTTGGGCTGTGGACTTATTAGTGTAAAAAAAATAATCATAATAAAAAAAAAGAGATAAAATCACCTTTTCTTTTAA

**Sequence 6 was used for designing RT-PCR primers for *EgFMO1***

XM_019853925.1 PREDICTED: Elaeis guineensis probable flavin-containing monooxygenase 1 (LOC105055196), mRNA AATTCGGACTTCTGTTTAACCCCAACCACTCGTTGGCCGATCTCATAAAATCTTTTCACAACCAATTTACCACTCGTTGGCCGATCTCATAAAATCTTTTCACAACCAATTTACCACTCCTTTTGTCCACTTCTTCGAAGATAATATCAAGAACCCTCCAGCTCATGGCTCAACTCCATTTATTTGCCACCCGGTAGTAAGAGGACACTGCACACCCTCTAGCCTCTAGGGGAAAGTTAGAGCGGAACTGCGCAAGAAAAGAAAGAGAGGAGGATGGAAAAGAAGAGAGTGGGCATAGTGGGGGCGGGCGTCAGTGGTCTGGTGGCGTGCAAGCACGCGTTGGAGAAAGGCTTCTTGCCGACCGTCTTCGAGGCCGAGGCCGACATCGGGGGAGTGTGGGCGCGCACGCTGGACTCAACGAAACTCCAGTCGCCGCGGCCGATCTACGAGTTCACGGACTTCCCGTGGCCAGCGAGCGTGACGGAGGCGGCGCCGGACCACCGGAAGGTGATGGACTACCTGCGGTCGTACGCCCGCCACTTCGACCTCCTTAGGTACGTCGAGTTCGAGGCGAGGGTGGTGGAAGTCGAGTACGTCGGGGCTAAGGAGGAGGAGATCATGGCGTGGGAGCTGTGGGCCGGAAACGGCGAGGCGTTCGGCGGTGGGCGGCGGGGGGAGTGGCACATCGCAGTGCAGAAAGGGGAGGATGGGTCCCCAGAGACATACCAAGTGGATTTTTTGATCCTTTGCATTGGGCGATTCAGCGGCACACCGAATATACCCACGTTTCCAGTAAACAAGGGTCCTGAAGTGTTTGATGGAAAGGTGATCCACTCCAGGGACTACTCAAACATGGGCAGTGCTCGCGCCACTGAACTGATTAAAGGAAAACGAGTTACAGTGGTAGGATTCCTGAAGTCAGCTTTGGACATTGCAGCAGAGTGCGCGAATGCAAATGGGGTGGAGTATCCATGTACGATGATATGTAGGACTAAGCGTTGGAACGTAGATGACTTCTATGTTTGGGGAATCCCTATAGCTTATTTCTATTTTAATCGATTCTCGGAGCTCCTTGTTCACAAGCCTGGTGAAGGATTTCTGCTTTGGTTATTGGCCATCCTGTTGTCACCTATTAGATGGTTGTTTTCTAAATTTGTTGAGAGCTACTATCGATGGGCAATTCCTATGAAGAGGTATGGTATGGTACCGGAGCATAGTTTCTTTCAAGCAATAACTTCTTGTTTGCTGGCAGTTCTGCCGGAAAAGTTTTATGATAGAGTGGAAGAAGGGAGCATTGTTCTCAAGCCATCAAAATCCTTTAGCTTCTGTAAGAATGGTGTGATCATCGAAGGTGAAGCAGCACCTATAAAGACTGATATACTGATCTTTGCCACTGGATTCAAGGGTGATCAGAAGCTCAGAGACATCTTTAGTTCACCTTTCTTTCAGAGGATTGTAGCCGGTTCATCAACTACGACCATCCCTCTCTATAGGGAGTGCATCCACCCCCGGATTCCACAATTGGCAATTCTTGGTTACTCGGAGAGCTTTACGAACTTACATACATCGGAGATGAGATCTAAGTGGCTATTTCATTTCCTCGATGGCGGTTTTCAACTCCCCAGCATAAGAGAAATGGAAAAGAATGTTTTAGAATGGGAGAAGTACATGAAACGATATTCTGGTGAGTATTACCGAAGATCTAGTATTGCAGCTCTCCATATTTGGTACAATGATCAGCTGTGCCGAGACATGGGATGCAACCCAAGAAGGAAGAAGGGATTCTTGGCTGACATGTTCTTTGCTTATGGACCTACAGATTATGCCAATCTTAACCCTAGCAAATAAGTGTTTTCAGCCATAACACATGGCGTATATAGCCTCATTCTCGAGGTGATCAAAGACCTAATTAGTTCTATTGATGTAACAAAGATTGTGGTGAGGCTTTGGACATGGTCTAGTTTCTGTTCTTGTATGTCTCCGTTGTCGATGAACAATGAGGAACACTGGGGGTACGTATGCA
