# Supplementary material for: Transcriptome analysis of oil palm inflorescences revealed candidate genes for an auxin signaling pathway involved in parthenocarpy
Source: PeerJ. 2018 Dec 17;6:e5975. doi: 10.7717/peerj.5975 (PMC6301279; doi:10.7717/peerj.5975)
Supplement: Supplemental Information 10 [file peerj-06-5975-s010.pdf]

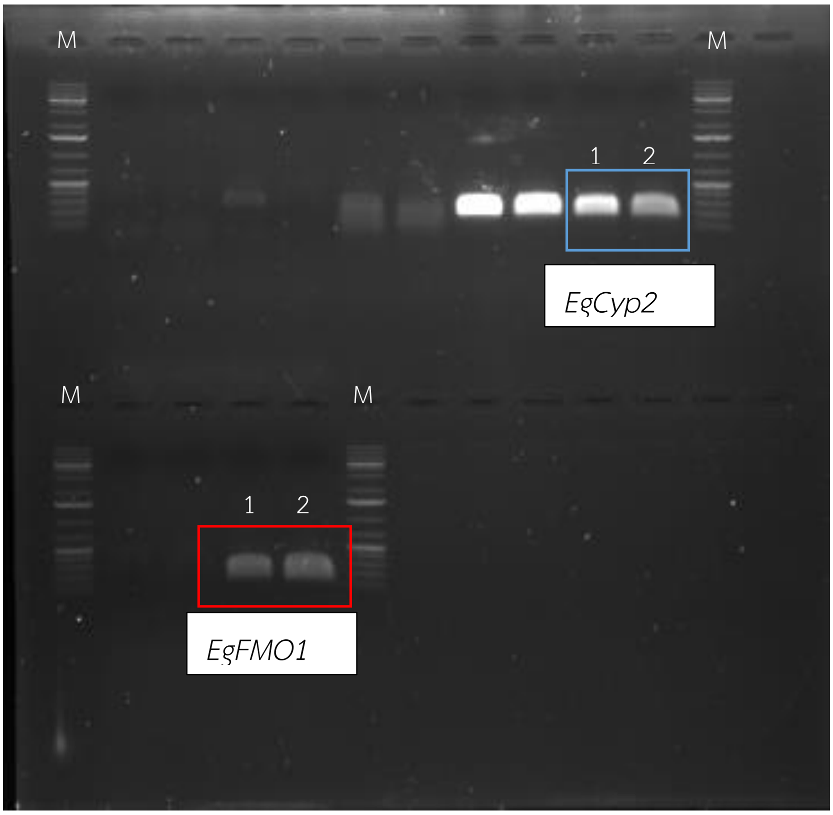

Original gel image 1 for Fig. 3A, the first RT-PCR comparison was between Inflo.1T/WA (Lane 1) and Inflo.1C/NA (Lane 2), by comparisons of *EgFMO1* amplification. *EgCyp2* was used as the reference gene. M = GeneRuler^{TM} 1 kb plus DNA ladder.

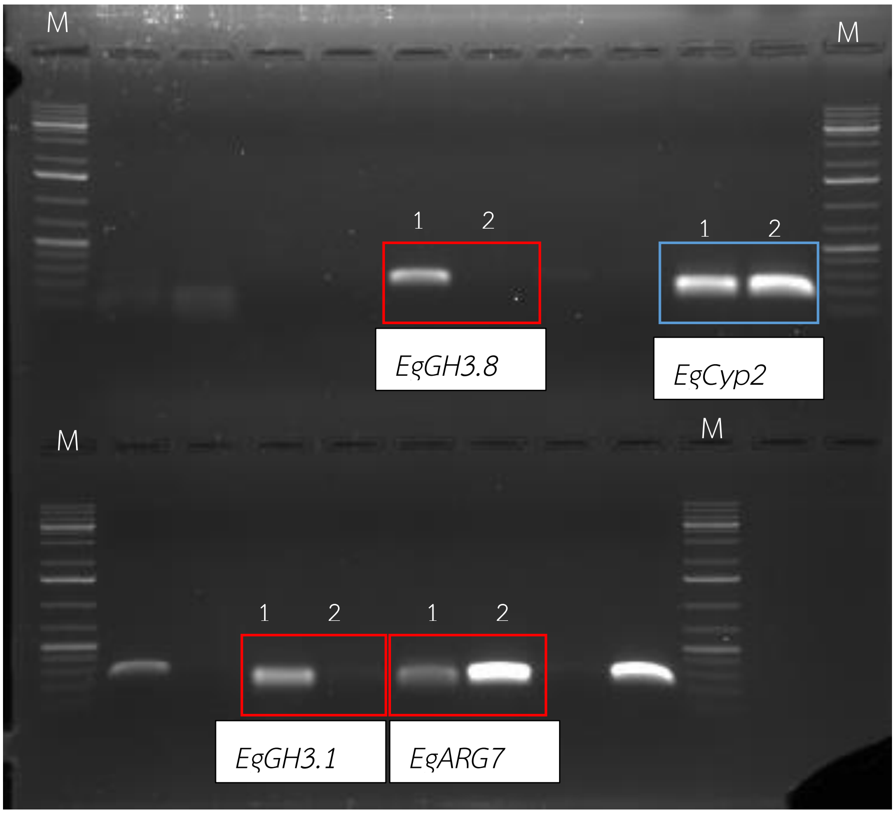

Original gel image 2 for Fig. 3B, the second RT-PCR comparison was between Inflo.6T/WA (Lane 1) and Inflo.2C/NA1 (Lane 2), by comparisons of *EgGH3.8*, *EgFH3.1* and *EgARG7* amplification. *EgCyp2* was used as the reference gene. M = GeneRuler™ 1 kb plus DNA ladder.

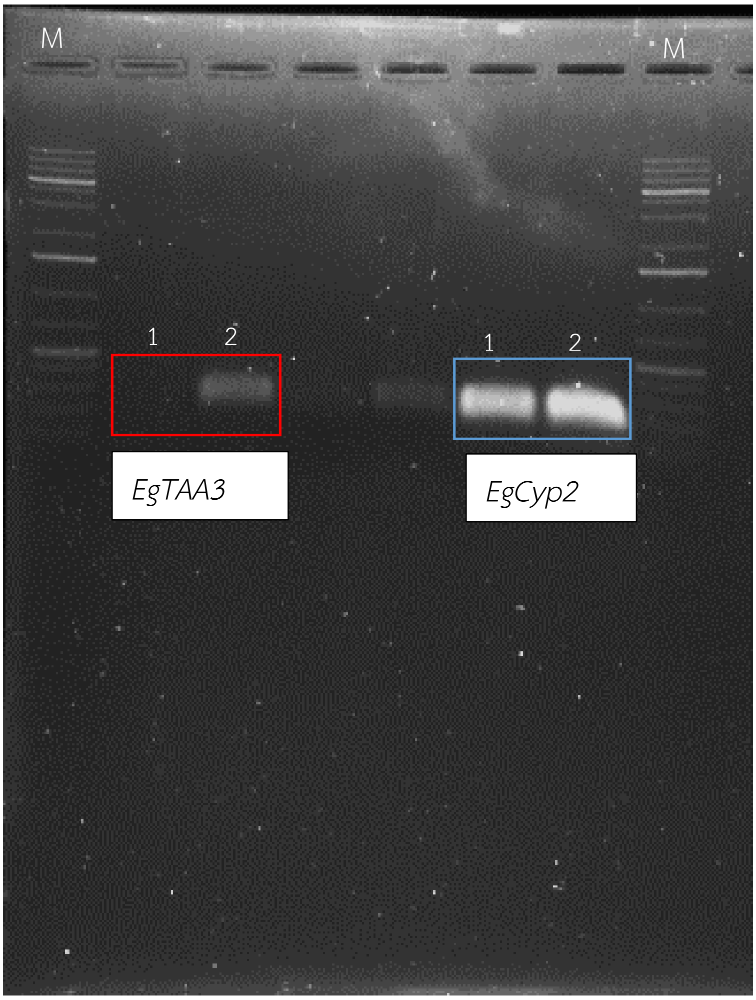

Original gel image 3 for Fig. 3C, the third RT-PCR comparison was between Inflo.6T/WA and inflo.4C/NA, by comparisons of *EgTAA3* amplification (C). *EgCyp2* was used as the reference gene. M = GeneRuler^{TM} 1 kb plus DNA ladder.
